# International Olympic Committee Sport Mental Health Assessment Tool 2 (IOC SMHAT2): development, content validity and feasibility

**DOI:** 10.1136/bjsports-2025-111408

**Published:** 2026-07-16

**Authors:** Vincent Gouttebarge, Abhinav Bindra, Cheri Blauwet, Alan Currie, Brian Hainline, Emily Kroshus-Havril, David McDuff, Rosemary Purcell, Margot Putukian, Claudia L Reardon, Simon M Rice, Scott Sloan, Jane Thornton, Margo Mountjoy

**Affiliations:** 1 Section Sports Medicine, Faculty of Health Sciences, University of Pretoria, Pretoria, South Africa; 2 Amsterdam UMC location University of Amsterdam, Department of Orthopedic Surgery and Sports Medicine, Amsterdam, Netherlands; 3 Athletes' Commission, International Olympic Committee, Lausanne, Switzerland; 4 Department of Physical Medicine and Rehabilitation, Shirley Ryan AbilityLab, Chicago, Illinois, USA; 5 Athlete Health, UK Sports Institute, Manchester, UK; 6 Department of Neurology, NYU Grossman School of Medicine, New York, New York, USA; 7 Department of Pediatrics, University of Washington, Seattle, Washington, USA; 8 Department of Psychiatry, University of Maryland School of Medicine, Baltimore, Maryland, USA; 9 Elite Sport and Mental Health, Orygen The National Centre of Excellence in Youth Mental Health, Parkville, Victoria, Australia; 10 Centre for Youth Mental Health, University of Melbourne, Melbourne, Victoria, Australia; 11 US Soccer Federation, Princeton University, Princeton, New Jersey, USA; 12 Department of Psychiatry, University of Wisconsin-Madison School of Medicine and Public Health, Madison, Wisconsin, USA; 13 Research and Translation, Orygen, The National Centre of Excellence in Youth Mental Health, Melbourne, Victoria, Australia; 14 Institute of Men’s Health, Movember, Richmond, Victoria, Australia; 15 Health, Medicine and Science, International Olympic Committee, Lausanne, Switzerland; 16 Western Centre for Public Health and Family Medicine, University of Western Ontario, London, Ontario, Canada; 17 Department of Family Medicine-Sport, McMaster University, Hamilton, Ontario, Canada

**Keywords:** Psychology, Sports, Sports medicine

## Abstract

**Objective:**

To describe the evidence- and practice-based processes that led to the International Olympic Committee Sport Mental Health Assessment Tool 2 (IOC SMHAT2).

**Methods:**

Seven stages were conducted including: (1) reviewing the scientific literature related to the use (eg, uptake by physicians) of the IOC SMHAT-1; (2) gathering the view (eg, experience with the tool, advice for improvements) of IOC SMHAT-1 users; (3) exploring the misclassification and measurement properties of the IOC SMHAT-1; (4) performing misclassification analysis of available IOC SMHAT-1 data; (5) designing the initial IOC SMHAT2; (6) evaluating the content validity and feasibility of the initial IOC SMHAT2; and (7) finalising the IOC SMHAT2.

**Results:**

The IOC SMHAT2 was developed for sports medicine physicians and other licensed/registered health professionals with the objective of identifying elite athletes (defined as professional, Olympic, Paralympic or collegiate level; 16 years of age and older) potentially experiencing mental health symptoms and disorders. The IOC SMHAT2 is comprised of a three-step approach: (1) universal screening as step 1, including five disorder-specific screening questionnaires and two single questions; (2) conditional screening as step 2, including two disorder-specific screening questionnaires; and (3) an aid to clinical assessment and treatment as step 3 that should be conducted by sports medicine physicians or licensed/registered mental health professionals.

**Conclusion:**

Based on scientific and anecdotal reflections on its first version, we developed the IOC SMHAT2 as a second updated version of the tool to identify elite athletes potentially experiencing mental health symptoms and disorders.

**WHAT IS ALREADY KNOWN ON THIS TOPIC**

**WHAT THIS STUDY ADDS**

- The IOC Sport Mental Health Assessment Tool 2 (IOC SMHAT2) was developed to identify elite athletes potentially experiencing mental health symptoms and disorders, in order to facilitate timely referral of those in need of support and/or treatment.
- The IOC SMHAT2 is comprised of a three-step approach including universal screening as step 1 (five disorder-specific screening questionnaires and two single questions), conditional screening as step 2 (two disorder-specific screening questionnaires) and an aid to clinical assessment (and treatment) as step 3.
- The IOC SMHAT2 can be used by sports medicine physicians and other licensed/registered health professionals, but further clinical assessment (and treatment) must be conducted by sports medicine physicians and/or licensed/registered mental health professionals, including psychiatrists and clinically trained psychologists.

**HOW THIS STUDY MIGHT AFFECT RESEARCH, PRACTICE OR POLICY**

- The IOC SMHAT2 should be embedded within the pre-season period (ie, ideally a few weeks after the start of sport training but before the competition period). The IOC SMHAT2 can also be used mid- and end-season or when any significant event for athletes occurs, such as injury, illness, surgery, any significant performance concern, after a major competition, end of competitive cycle, suspected harassment/abuse, adverse life event, deselection and/or transitioning out of sport.
- Future validation studies of the IOC SMHAT2 should be continued among elite athletes from various sport disciplines and cultures.

## Introduction

 Mental health symptoms and disorders, including those involving anxiety, depression, substance misuse and disordered eating, are common problems among elite athletes, with prevalence rates similar to or greater than those in the general population.^1–3^ In 2019, following the recommendations made in the *Internaitonal Olympic Committee (IOC) consensus statement on mental health in elite athletes*, the IOC Mental Health Working Group (MHWG) developed the IOC Sport Mental Health Assessment Tool 1 (SMHAT-1).^4 5^ The IOC SMHAT-1 was designed to be used by sports medicine physicians and other licensed/registered health professionals to screen elite athletes (defined as professional, Olympic, Paralympic or collegiate level; aged 16 years and older) potentially at risk for, or already experiencing, mental health symptoms or disorders.^5^ The ultimate intention of the IOC SMHAT-1 was to facilitate timely identification, management and/or treatment of mental health symptoms and disorders.^5^ Since its development, the IOC SMHAT-1 has been used in various research and clinical settings.^6–9^ This has raised some concerns among others with regard to the reliance on self-reported data as well as with the potential for subjectivity and under- or over-reporting of symptoms.^6–9^ This has also led to scientific and practice-related directions for further development and potential improvements to enhance feasibility, reliability and validity. This article describes the evidence- and practice-based process that led to the second version of the tool, namely the IOC Sport Mental Health Assessment Tool 2 (IOC SMHAT2).

## Methods

A mixed-methods study was conducted to reflect on and refine the first version of the tool, to produce the IOC SMHAT2. Guided by COSMIN methodology,^10^ our study was based on five development (stage 1 to stage 5) and two validation (stage 6 and stage 7) stages, namely: (stage 1) reviewing the scientific literature related to the use (eg, uptake by clinicians) of the IOC SMHAT-1; (stage 2) conducting interviews with experienced users of the IOC SMHAT-1; (stage 3) reviewing the scientific literature related to misclassification and measurement properties of the IOC SMHAT-1; (stage 4) performing misclassification analysis of available data collected with the IOC SMHAT-1; (stage 5) convening an expert meeting to design an initial IOC SMHAT2; (stage 6) exploring the content validity and feasibility of the initial IOC SMHAT2; (stage 7) designing and finalising the IOC SMHAT2.

### Stage 1 (development): scientific literature related to the use of the IOC SMHAT-1

A scoping review of the scientific literature was conducted to identify the potential strengths and limitations of the IOC SMHAT-1. The electronic databases MEDLINE (via PubMed), SPORTDiscus (via EBSCO) and PsycINFO (via EBSCO) were searched from 2020 (year of publication of the IOC SMHAT-1) to May 2025, applying the following filters (if applicable): humans; English; academic journal; peer-reviewed journal. A sensitive search strategy was built based on two search terms, namely ‘Sport Mental Health Assessment Tool’ and ‘SMHAT’. Both search terms were combined by the Boolean command OR. Eligibility criteria for inclusion were:

- The study population consisted exclusively of active or former elite athletes (professional, Olympic, Paralympic or collegiate level).
- The article presented an original study.
- The study used the IOC SMHAT-1 to assess mental health symptoms or disorders.
- The article was written in English.

To identify potentially relevant articles, full-text articles were screened by one researcher (VG) using the eligibility criteria and verified independently by a second researcher (MM). To avoid missing any relevant publications, the references of the included studies were also screened, as well as potentially relevant preprints. Disagreements regarding the inclusion or exclusion of articles were resolved via consensus between both researchers (VG, MM), involving a third researcher (RP) if consensus was not reached.

Data from the included articles were extracted by one researcher (VG) and verified independently by a second researcher (MM). A standardised extraction form was used in order to report the following: study information (author, year); study population (eg, sample size, age, country, level of competition); study design and related information if applicable (eg, response rate, follow-up period); qualitative information about the strengths and limitations of the IOC SMHAT-1 as identified by the respective author(s).

### Stage 2 (development): interviews with experienced users of the IOC SMHAT-1

A qualitative study utilising individual online semi-structured interviews with experienced users of the IOC SMHAT-1 was conducted to gauge their experiences with the tool, to reflect on its principal components and related constructs (ie, sport-related psychological distress, depression, anxiety, sleep disturbance, alcohol misuse, substance misuse, disordered eating) and to gather their advice for improvements in the second version. Formal ethical approval was not needed as this study did not meet institutional criteria for the Medical Research Involving Human Subjects Act.

Participants were experienced users of the IOC SMHAT-1 fulfilling the following inclusion criteria: (i) being 18 years and older, (ii) able to converse in English and (iii) having administered the IOC SMHAT-1 in a research and/or clinical setting (eg, during sporting events, team or school-based care). Potential participants were identified via the existing literature related to the IOC SMHAT-1 (eg, first author of a published article focusing on the use of the IOC SMHAT-1) as well as via an email within the network of the IOC and its MHWG. This recruitment approach was applied to obtain global experience as was feasible. A sample size calculation was not performed as we anticipated that a limited number of experienced users would be available (based on the IOC’s network and the existing literature as collected in step 1).

The interview form was developed by the IOC and its MHWG, including six questions that explored the context in which the participants used the IOC SMHAT-1, the facilitators and barriers experienced by participants in the use of the IOC SMHAT-1 and the view of participants on the three steps of the IOC SMHAT-1 (online supplemental material 1). For each question, participants were able to fully expand and provide their experiences in the use of the IOC SMHAT-1 and their recommendations for the IOC SMHAT2.

Interviews were conducted online by two researchers (VG, MM) using the digital platform Microsoft Teams. All interviews were conducted between January and April 2025, lasting on average 20 min. Participants received information about the interviews in advance and consented to participate via email. Prior to progressing to the first question of the interview, participants were provided with the study overview and objectives. They were also asked to provide their consent for recording the interview. At the conclusion, participants were thanked and asked if they had any further comments they would like to share.

The recorded interviews were transcribed with the online artificial intelligence transcription service Rev Artificial Intelligence (www.rev.com). The transcripts were checked by two researchers (VG, MM) and subsequently by the participants to verify the competency of the software in transcribing accurately as well as to confirm that no relevant information was missing. Then, the transcripts were initially analysed with the Artificial Intelligence software ATLAS.ti for Mac (www.atlasti.com) by a thematic analysis, using a semantic approach.^11^ The automatically generated patterns and themes were subsequently reviewed and refined by the researchers to ensure accuracy and validity before completing the thematic analysis. During this process, data saturation was assessed through iterative analysis conducted alongside data collection. Data saturation was established once three consecutive interviews did not provide any new insights and themes. All transcripts were read multiple times and themes were formed by identifying recurring patterns of views explicitly stated by the participants. All views were summarised for each theme, striving to describe shared experiences across participants.

### Stage 3 (development): scientific literature related to misclassification and measurement properties of the IOC SMHAT-1

A scoping review of the scientific literature was conducted to explore the potential misclassification (eg, false negative) between step 1, step 2 and/or step 3 (eg, negative on step 1 but positive on step 2) and measurement properties (eg, internal consistency) of the IOC SMHAT-1. Therefore, the same approach as in stage 1 was used (eg, sensitive search strategy, three electronic databases). Eligibility criteria for inclusion were:

- The article presented an original quantitative study.
- The study outcomes related to misclassification and/or measurement properties.
- The article was written in English.

To identify potentially relevant articles, full text articles were screened by one researcher (VG) using the eligibility criteria and verified independently by a second researcher (RP). To avoid missing any relevant publications, the references of the included studies were screened as well. Any disagreements regarding the inclusion or exclusion of articles were resolved via consensus. Using the same standardised extraction form from stage 1, data from the included articles were extracted by one researcher (VG) and verified independently by a second researcher (RP).

### Stage 4 (development): misclassification analysis of available data collected with the IOC SMHAT-1

To complement the information on misclassifications of the IOC SMHAT-1 gathered through the existing literature (stage 3), various analyses were performed on original datasets previously collected with the IOC SMHAT-1 by members of the IOC MHWG among elite athletes.^7 8 12–14^ The nature of these analyses was mostly based on the information gathered from the literature (stage 1 and stage 3) and from the experiences and advice of users (stage 2). Analysis of misclassification between step 1 and step 2 of the IOC SMHAT-1 (eg, false negatives in the form of negative on step 1 but positive on step 2) was performed with crosstabulation across all included questionnaires, namely, the Athlete Psychological Strain Questionnaire (APSQ), Generalised Anxiety Disorder 7-item (GAD-7), Patient Health Questionnaire 9-item (PHQ-9), Athlete Sleep Screening Questionnaire (ASSQ), Alcohol Use Disorder Identification Test-Consumption (AUDIT-C), Cut down Annoyed Guilty Eye opener Adapted to Include Drugs (CAGE-AID) and Brief Eating Disorder in Athletes Questionnaire (BEDA-Q). Furthermore, concurrent validity of the shorter version Generalised Anxiety Disorder 2-item (GAD-2) and Patient Health Questionnaire 2-item (PHQ-2) with their respective longer version GAD-7 and PHQ-9 was explored with crosstabulation, presenting the false negative (FNR) and false positive (FPR) rates between both versions.

### Stage 5 (development): expert meeting to design the initial IOC SMHAT2

A 2-day expert meeting was held in June 2025 in Lausanne, Switzerland, at Olympic House, during which all information gathered in the previous stages was presented and discussed. Participants of the meeting (most of whom were members of the IOC MHWG) were international experts with specialised training, skills and experience in a wide range of relevant domains, including but not limited to psychiatry, sports medicine, sports neurology, addiction medicine, measurement properties and elite sport itself. The chair of the IOC MHWG (VG) coordinated and facilitated the expert meeting, ensuring a structured discussion and balanced contributions from all participating experts. The discussion was guided to critically reflect on the existing evidence gathered in the previous stages, methodological options and practical aspects relevant to the tool’s objective. Expert feedback was verbally synthesised and analysed to identify the flow and key components of the tool. Through the meeting, progressive refinement of the tool was reached through discussion and clarification of divergent points of view, striving to reach alignment on the flow and key components of the tool. This process informed the development and design of the initial IOC SMHAT2.

### Stage 6 (validation): content validity and feasibility of the initial IOC SMHAT2

Mixed-methods feedback was sought from intended end users (healthcare professionals) about the content validity and feasibility of the initial IOC SMHAT2. Content validation is typically explored during a measure’s development, where experts rate initial items in terms of their relevance, comprehensiveness and comprehensibility for assessing the construct of interest in the target population and setting.^10^ Because the IOC SMHAT2 is composed of existing validated questionnaires that are not subject to potential item-level modification, we sought feedback on the relevance of including specific screening steps and questionnaires, and on guidance for tool implementation.

Healthcare professionals (eg, sports medicine physicians, psychologists) were recruited as participants. Study information was shared by IOC MHWG via email to the members of the Association of Summer Olympic International Federations and the Winter Olympic Federations. A snowball sampling approach was also used to distribute study information to healthcare professionals who provide mental healthcare to elite athletes through the networks of the IOC MHWG. Potential participants reviewed a study information sheet, indicated their willingness to participate, and were subsequently sent an anonymous online survey hosted on the Qualtrics survey platform.

First, participants were asked to review the full IOC SMHAT2, which was included as an attachment in the online survey. Next, they were presented with screenshots of each component of the initial IOC SMHAT2 (eg, introductory text, screening steps, clinical assessment), with accompanying questions about that component’s relevance and appropriateness. Each component, including each screening questionnaire, was queried with a single question (eg, ‘Is this component relevant and appropriate for use with elite athletes?’), with response options ranging from ‘strongly disagree’ (1) to ‘strongly agree’ (9), a rating approach commonly used in Delphi-based consensus processes.^15–17^ Opportunity for open-ended feedback was provided for each component and encouraged for any scores of 6 or less. After completing component-level ratings, participants were asked to provide similarly scored assessments of comprehensiveness and comprehensibility, along with open-ended feedback. Participants were asked to complete the 4-item Feasibility of Intervention Measure (FIM) to explore the feasibility of the initial IOC SMHAT2.^18^ Item-level responses ranging from ‘completely disagree’ (1) to ‘completely agree’ (5) are summed to create an overall score ranging from 4 to 20, with higher scores indicating greater feasibility. Respondents were also encouraged to share open-ended feedback about feasibility.

Means and measures of dispersion were calculated for the full IOC SMHAT2 and for each component. A priori, we established thresholds for component-level ratings of at least 70% of respondents scoring components as 7, 8 or 9. This is among the most frequently used numeric thresholds for determining consensus when a 1–9 rating scale is used in Delphi-based consensus approaches.^19^ Scores on the FIM were reviewed using a score of >16 as a reference point for feasibility.^18^ Open-ended comments about feasibility and for components not meeting a priori thresholds for appropriateness and relevance were reviewed using a pragmatic application of descriptive content analytic methods,^20^ with the goal of summarising response patterns. After data familiarisation, comments addressing similar topics were grouped together by one researcher (EK-H). This was shared with the IOC MHWG for iterative discussion and refinement. The output of this process was used to identify any possible modifications to the tool itself, as well as recurrent concerns and suggestions about feasibility. If any changes were made to the IOC SMHAT2 as a result of this process, the revised tool was re-sent to participants for re-rating using the mixed-methods online survey process described.

### Stage 7 (validation): designing and finalising the IOC SMHAT2

A meeting of the IOC MHWG was held online via Microsoft Teams (Microsoft Corporation, Redmond, WA, USA) in December 2025. During that meeting led by the chair of the IOC MHWG (VG), the quantitative and qualitative feedback provided by medical professionals on the content validity and feasibility of the initial IOC SMHAT2 (stage 6) was discussed. Subsequently, the IOC SMHAT2 was finalised.

### Patient and public involvement

The IOC MHWG that designed this mixed-methods study included both medical professionals (ie, sports medicine physicians, psychiatrists, psychologists) and (former) elite athletes, which is most relevant in the development of the IOC SMHAT2 (stages 5 and 7). Healthcare professionals (eg, sports medicine physicians, psychologists) were involved in reflecting on and refining the first version of the tool (stage 2), as well as in developing and designing the second version of the tool (stage 6).

### Equity, diversity and inclusion

The authors’ group is gender balanced and consists of clinicians and researchers from different health-related disciplines. The authors come from various countries across several continents and are representative of individuals of diverse racial and ethnic backgrounds as well as the disability community. The study population from our analyses (stage 4) included men and women athletes from low-, middle- and high-income countries and from various sports.

## Results

### Scientific literature related to the use of the IOC SMHAT-1 (stage 1)

**Figure 1:**
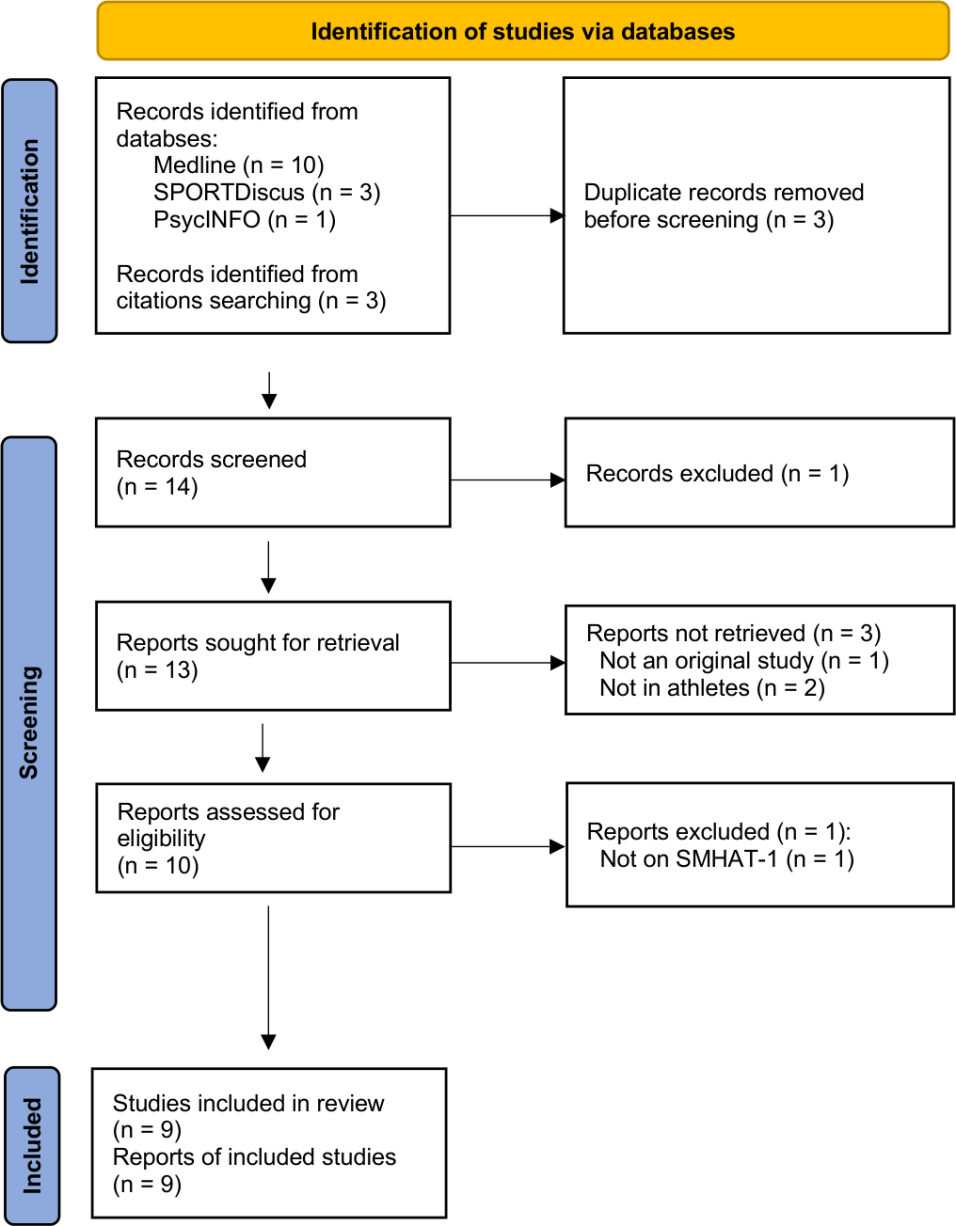
Preferred Reporting Items for Systematic Reviews and Meta-Analyses flow diagram of literature search (stage 1 and stage 3). SMHAT-1, Sport Mental Health Assessment Tool 1.

A total of 14 potentially relevant studies were retrieved from the databases. Three additional studies were identified via reference list screening, while three duplicates were removed. After applying the inclusion criteria to the titles, abstracts and/or full text, five studies were excluded because they were either not original studies or not conducted in elite athletes. Consequently, nine original studies were ultimately included (figure 1).^7–9 12 13 21–24^

The included studies provided limited qualitative insights into the strengths and limitations of the IOC SMHAT-1 (online supplemental material 2). An acknowledged strength was its standardised approach, which effectively detected a broad range of mental health symptoms, raised awareness and supported early identification and intervention. Step 1, conducted via the APSQ, showed some utility but required improvement due to notable FNR and FPR, raising concerns about its reliability as a triage measure for a broad range of mental health symptom clusters in elite athletes. The APSQ could be quickly completed and demonstrated high accuracy in detecting concerns such as suicidality, sleep disturbance, substance misuse and disordered eating. Some concerns were raised regarding the reliance of self-reported data, and the potential for subjectivity and under- or over-reporting of symptoms. Several thresholds in steps 1 and 2 (eg, APSQ, BEDA-Q) required further validation, highlighting the importance of the clinical interview in step 3 for a comprehensive assessment of an athlete’s mental health.

### Interviews with experienced users (stage 2)

Interviews with eight experienced users were conducted (data saturation was reached after the sixth). All interviewees were mental health professionals (six psychologists, two sports medicine physicians, one psychiatrist) and had administered the IOC SMHAT-1 to elite athletes (defined as professional, Olympic, Paralympic or collegiate level; aged 16 years and older) in combined research and clinical settings. Several facilitators and barriers for the use of the IOC SMHAT-1 were stated during the interviews (table 1). Principally, facilitators, including digital availability of the tool would enhance its usability and efficiency, while providing feedback to athletes remained essential. Once a sport organisation endorses its use and after staff member training, the IOC SMHAT-1 should be integrated in the regular health screening process. This practice would serve to normalise its use and reduce stigma. In contrast, time constraints, limited resources (eg, lack of referral process), confidentiality concerns, as well as cultural and language challenges, were stated as potential barriers for the use of the IOC SMHAT-1. One participant stated, “Although athletes were informed in advance that the questionnaire would take 15–20 min, the length may still have posed a practical barrier for some of them. However, the time requirement was clear and manageable for most of them”. Another barrier mentioned by the interviewees for the use of the IOC SMHAT-1 was the timing of its administration (eg, during busy pre-season) and its availability in paper-based format only and not in electronic format.

**Table 1:** Summary of the interviews with experienced users of the IOC Sport Mental Health Assessment Tool 1 (SMHAT-1)

Facilitators	Barriers	Value of step 1 and step 2	Missing questionnaire(s)	Clinical assessment (step 3)
Digital availability: a digital version of SMHAT-1 would enhance accessibility, usability and efficiency	Time constraints: both athletes and staff might find the process time-consuming, especially step 2	Step 1 (APSQ): quick, easy to administer but concerns about reliability due to false positives/negatives Step 1 (APSQ): some users questioned its value, others found it useful for broad screening	Trauma-related construct: highlighted by multiple users as a major omission, especially considering its relevance in athlete mental health	Essential component: critical for confirming or clarifying findings from step 1 and step 2 as well as for making clinical decisions. Essential component: structured clinical judgement is needed for accurate interpretation
Organisational endorsement and support: adoption and support by sports bodies increases legitimacy and implementation	Limited resources: lack of trained personnel and weak referral pathways reduce effectiveness and follow-up care	Step 2: valued for its structured and evidence-based format Step 2: time-intensive but users appreciated its thoroughness with all six key mental health symptom clusters seen as relevant	Cultural sensitivity tools: suggested to better address language and cultural barriers in diverse sporting populations	Template and guidance needs: users, especially those without a mental health background, requested more standardised templates, clearer instructions and examples
Training of staff: training improves proper usage, scoring, interpretation and athlete communication	Confidentiality issues: athletes may fear breaches of privacy or stigma, discouraging honest responses	The stepwise approach (3-step process) was seen as a strength, offering a clear flow from screening to clinical action	No standard grief/loss or burnout screening tools while some users mentioned these might be useful in athlete populations	Multidisciplinary support: integration with medical and mental health professionals is seen as vital for accurate follow-up and treatment
Integration into health screenings: this routine use would normalise mental health screening, increase awareness and reduce stigma	Cultural and language barriers: some questions might not be fully adapted for non-native speakers (not validated in all languages) or different cultural contexts	Mixed views on removing step 1: some would prefer going directly to step 2 due to APSQ limitations, while others value it for early triage Mixed views on removing step 1: no consensus among users		Clear clinical guidelines and decision-making pathways are especially important for non-specialists who are often the first line of contact in sports settings
Feedback to athletes: providing results or support feedback fosters engagement and perceived value	Paper-based format limitations: the current paper format is less efficient, and digitisation would improve data handling and reduce burden			Clinical assessments are viewed as the most accurate, but they require time and clinical training to implement effectively

APSQ, Athlete Psychological Strain Questionnaire.

Experienced users mentioned the importance of the structured, evidence-based and stepwise approach of the IOC SMHAT-1. One participant stated, “The three-step approach, the stepwise approach is a strength as we like to follow protocol. It provides a clear and logical progression from initial screening to clinical assessment and informed clinical action”. While step 2 is thorough but time-consuming, some concerns were flagged regarding the false positives/negatives associated with step 1 (APSQ). However, no consensus could be identified among experienced users about the inclusion or exclusion of this first step. Within step 2, the six included mental health symptom clusters were deemed to be relevant to experienced users. The trauma-related construct was repeatedly identified by experienced users as a potential addition to step 2. The clinical assessment (step 3) was found to be crucial for interpreting symptoms and ensuring appropriate management, but experienced users also mentioned that more guidance with clearer instructions on structured templates would be of added value, especially for non-mental health professionals (eg, health professionals without specific mental health expertise/training). One participant stated, “For our team, the approach in step 3 offered sufficient flexibility while still providing formalised structure through guiding questions, and clear referral points when needed”.

### Scientific literature related to misclassification and measurement properties of the IOC SMHAT-1 (stage 3)

Five studies explored the misclassification and measurement properties of the IOC SMHAT-1 (online supplemental material 2).^7 9 21 23 24^ The FNR of step 1 (triage) versus step 2 (extensive screening) ranged between 0% (anxiety, depression) and more than 60% (alcohol misuse, disordered eating), while the FPR of step 1 versus step 2 reached more than 60% for most of the mental health symptom clusters. The FNR of step 1 (triage) versus step 3 (clinical interview) was 10% for recommended action and 1% for referral, while the FPR of step 1 versus step 3 was 64% for recommended action and 70% for referral.

### Misclassification analysis of available data (stage 4)

Peer-reviewed data representing a total of 1035 elite athletes (mean age: 24 years; 55% men; 45% women) were available for misclassification analyses (table 2).^7 8 12–14^ The FNR of step 1 (triage with APSQ) versus step 2 (extensive screening) was 1% for GAD-7, 2% for PHQ-9, 23% for ASSQ, 35% for AUDIT-C, 21% for CAGE-AID and 34% for BEDA-Q. The FPR of step 1 (triage with APSQ) versus step 2 (extensive screening) was 54% for GAD-7, 53% for PHQ-9, 52% for ASSQ, 53% for AUDIT-C, 57% for CAGE-AID and 52% for BEDA-Q. Crosstabulations between the GAD-2 and the GAD-7 revealed that the FNR was 18% and the FPR was 5%. The FNR between the PHQ-2 and PHQ-9 was 38% and the FPR was 1%.

**Table 2:** Misclassification of the IOC Sport Mental Health Assessment Tool 1 (SMHAT-1) among elite athletes

		APSQ			GAD-2			PHQ-2		
		<17	≥17		<3	≥3		<3	≥3	
GAD-7	<10	431	512	FNR=1.4	893	45	FNR=18.1			
	≥10	1	71	FPR=54.3	13	59	FPR=4.8			
PHQ-9	<10	427	487	FNR=2.2				900	13	FNR=37.6
	≥10	2	91	FPR=53.3				35	58	FPR=1.4
ASSQ	<8	374	404	FNR=22.7						
	≥8	50	170	FPR=51.9						
AUDIT-C	<4 (M) 3 (W)	303	341	FNR=35.3						
	≥4 (M) 3 (W)	134	246	FPR=53.0						
CAGE-AID	<2	418	555	FNR=21.1						
	≥2	4	15	FPR=57.0						
BEDA-Q	<4	304	334	FNR=33.5						
	≥4	119	236	FPR=52.4						

APSQ, Athlete Psychological Strain Questionnaire; ASSQ, Athlete Sleep Screening Questionnaire; AUDIT-C, Alcohol Use Disorder Identification Test-Consumption; BEDA-Q, Brief Eating Disorder in Athletes Questionnaire; CAGE-AID, Cut down Annoyed Guilty Eye opener Adapted to Include Drugs; FNR, false negative rate; FPR, false positive rate; GAD-2, Generalised Anxiety Disorder 2-item; GAD-7, Generalised Anxiety Disorder 7-item; PHQ-2, Patient Health Questionnaire 2-item; PHQ-9, Patient Health Questionnaire 9-item.

### Initial IOC SMHAT2: development, validity and feasibility (stage 5 and stage 6)

Based on all information gathered through stages 1 to 4, experts decided to keep a three-step approach for the IOC SMHAT2, including universal screening as step 1, conditional screening as step 2, and an aid to clinical assessment (and treatment) as step 3. Furthermore, experts opted to redefine the flow of the IOC SMHAT-1 (eg, removal of the APSQ as a triage step), while some of the existing validated screening questionnaires originally selected for the IOC SMHAT-1 were changed.

A total of 58 healthcare professionals (39% Europe; 38% North America; 14% Asia; 9% Africa) completed the survey related to the content validity and feasibility of the initial IOC SMHAT2. Most of the participants (53% men; 47% women) were sports medicine physicians (41%) working clinically with elite athletes for 11 years on average (range 2–20 years) and having used the IOC SMHAT-1 previously (67%). All participant characteristics are presented in table 3.

**Table 3:** Participants’ characteristics (N=58)

Gender (%)	Men: 52.7	Women: 47.3
Region (%)		
Africa	8.9	
Asia	14.3	
Europe	39.3	
North America	37.5	
Oceania/Australia	0	
South America	0	
Professional role (%)		
Sports medicine physician	41.1	
Psychiatrist	8.9	
Other medical doctor	8.9	
Clinical or counselling psychologist	17.9	
Social worker	1.8	
Nurse	1.8	
Sport psychologist	10.7	
Other role	8.9	
Type of athletes under care* (%)		
Youth athletes (<18 years of age)	48.3	
College athletes	50.0	
Professional athletes	69.0	
Olympic sport athletes	72.4	
Working experience with elite athletes (in years; mean±SD; range)	11.3	±6.7 (2–20)
Previously used on the IOC SMHAT-1 (%)	66.7	

* Multiple responses allowed.

IOC SMHAT-1, IOC Sport Mental Health Assessment Tool 1.

Predetermined criteria (ie, more than 70% of responses for each component being rated as 7 or greater) were met for the relevance and appropriateness of the content of the IOC SMHAT2 (table 4). Mean ratings for each component of the IOC SMHAT2 were also greater than 7, indicating that on average participants ‘agreed’ or ‘strongly agreed’ that the respective components were relevant and appropriate for use with elite athletes. Scores for the Form 1 cannabis use and nicotine/tobacco use screeners were the lowest and had the most variance, with one quarter of the participants providing ratings of less than 7. However, both screeners did meet predetermined thresholds for continued inclusion without modification. On average, the IOC SMHAT2 was rated as being feasible, with notable variance. The sample mean score on FIM was 15.8 (SD=2.9), corresponding to an item-level mean of ‘agreeing’ that the IOC SMHAT2 was feasible. Two-thirds (68%) of the participants had a FIM score of greater than 15.

**Table 4:** Ratings of relevance and appropriateness of the IOC SMHAT2 components

Step	Component	Mean±SD	%
Step 1	Form 1, Screening 1 (anxiety)	8.5±1.0	94.8
Universal screening	Form 1, Screening 2 (depression and suicidal ideation)	8.3±1.0	93.1
	Form 1, Screening 3 (cannabis use)	7.2±2.2	74.1
	Form 1, Screening 4 (nicotine/tobacco use)	7.3±2.2	74.1
	Form 1, Screening 5 (sleep disturbance)	8.3±1.1	93.0
	Form 1, Screening 6 (disordered eating)	8.2±1.1	92.9
	Form 1, Screening 7 (alcohol misuse)	7.6±1.8	85.7
Step 2	Form 2, Screening 8 (post-traumatic stress)	8.0±1.3	87.9
Conditional screening	Form 2, Screening 9 (substance misuse)	7.7±1.7	84.5
Step 3	Guidance about clinical assessment and treatment	7.7±1.7	86.2
Clinical assessment and treatment	Form 3, Screening 10 (attention-deficit/hyperactivity disorder)	7.7±1.7	82.8
	Form 3, Screening 11 (bipolar disorder)	7.6±1.9	84.5
	Form 3, Screening 12 (problematic gambling)	7.9±1.7	89.5
	Form 3, Screening 13 (prolonged grief)	7.6±1.8	80.7
	Form 3, Screening 14 (obsessive-compulsive disorder)	7.7±1.7	86.0
	Form 3, Screening 15 (psychosis)	7.4±2.1	77.2

Participants also provided qualitative feedback on the tool (online supplemental material 3). The most frequently mentioned determinant of feasibility was resources. Some participants noted that it was implementable in their setting because of their resources, while others noted that clinical settings with fewer resources would have more difficulty with implementation. Suggested ways to increase feasibility included having automated administration and scoring, ensuring it was translated into multiple languages and considering an even briefer initial screening. Participants also raised feasibility concerns about Form 3 specifically. This included some confusion over the grid with instructions for denoting severity and complexity. Several participants also expressed a belief that step 3 should be implemented by a licensed mental health professional and noted that it was not clear to them from the written guidance that this was the case.

### Description of the IOC SMHAT2 (stage 7)

Because of the outcomes related to the content validity and feasibility of the initial IOC SMHAT2 (stage 6), no significant alterations of the IOC SMHAT2 were made (one typo corrected). Therefore, the IOC MHWG, following review and deliberation, was able to finalise the IOC SMHAT2.

#### Approach, objective and target group

**Figure 2:**
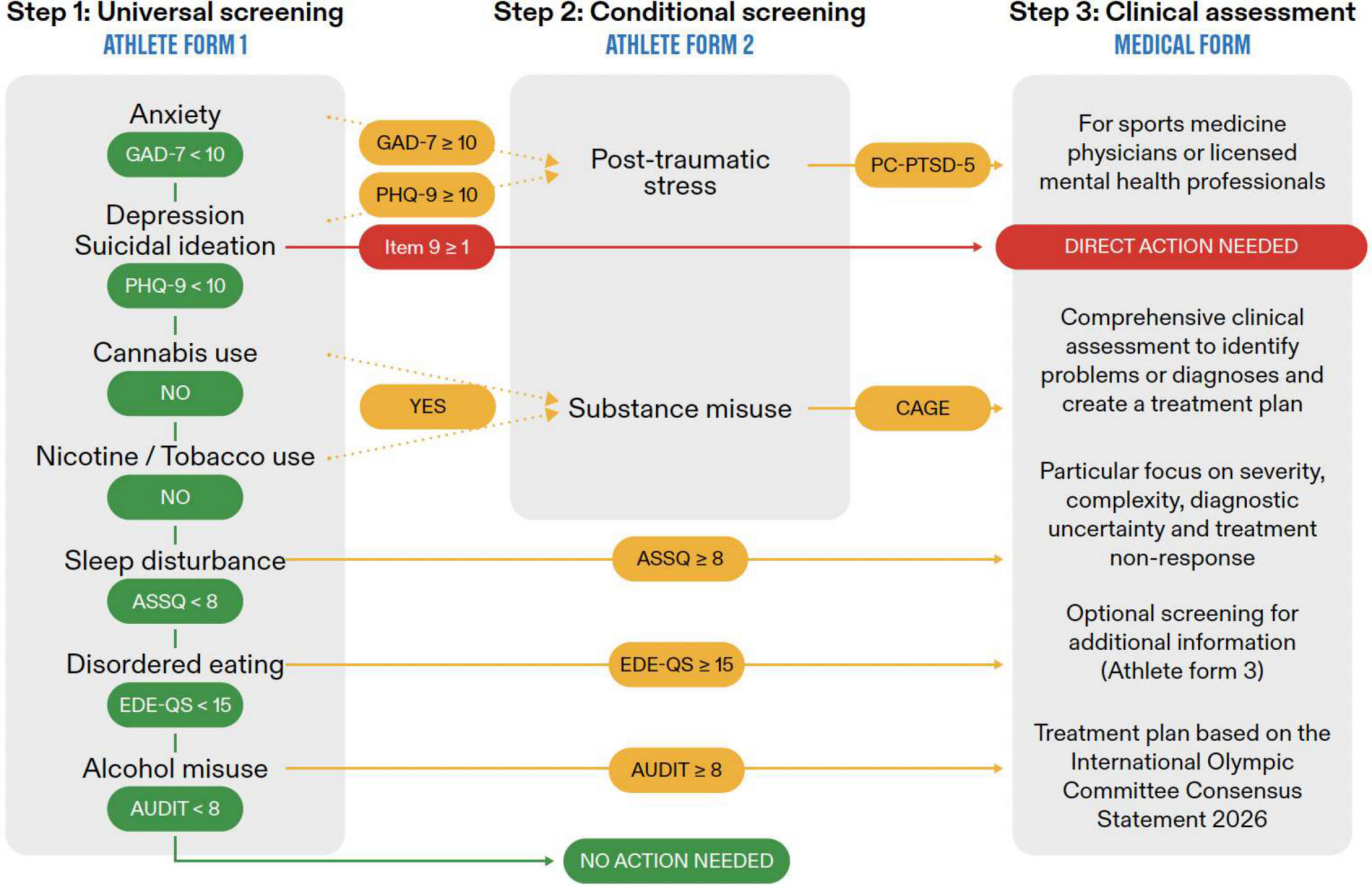
Flow of the IOC Sport Mental Health Assessment Tool 2 (IOC SMHAT2). ASSQ, Athlete Sleep Screening Questionnaire; AUDIT, Alcohol Use Disorder Identification Test; CAGE, Cut down Annoyed Guilty Eye opener; EDE-QS, Eating Disorder Examination Questionnaire Short; GAD-7, Generalised Anxiety Disorder 7-item; PC-PTSD-5, Primary Care Post Traumatic Stress Disorder Screen for DSM-5; PHQ-9, Patient Health Questionnaire 9-item.

Through a three-step approach including universal screening as step 1, conditional screening as step 2 and an aid to clinical assessment (and treatment) as step 3 (figure 2), the IOC SMHAT2 aims to identify elite athletes (defined as professional, Olympic, Paralympic or collegiate level; 16 years of age and older) potentially experiencing mental health symptoms and disorders, in order to facilitate timely referral of those in need of support and/or treatment (online supplemental material 4). The IOC SMHAT2 can be used by sports medicine physicians and other licensed/registered health professionals, but the clinical assessment (and related treatment) within the IOC SMHAT2 (see step 3) must be conducted by sports medicine physicians and/or licensed/registered mental health professionals, including clinically trained psychologists. Physical therapists, athletic trainers and psychologists without clinical training working with a sports medicine physician can use the IOC SMHAT2, but any diagnosis, guidance or intervention should remain the responsibility of the sports medicine physician and/or licensed/registered mental health professional.

#### Step 1: universal screening

The universal screening step includes five disorder-specific screening questionnaires and two single questions (online supplemental material 5), namely:

- GAD-7 assessing the presence of symptoms of anxiety over the last 2 weeks.^25^
- PHQ-9 assessing the presence of symptoms of depression over the last 2 weeks.^26 27^
- ASSQ assessing the presence of sleep disturbance over the recent past.^28–30^
- Eating Disorder Examination Questionnaire Short (EDE-QS) assessing the presence of disordered eating over the past 7 days.^31 32^
- AUDIT assessing the presence of alcohol misuse over the past year.^33 34^
- Single question assessing the use of cannabis/marijuana-containing products or related products in the last 6 months.
- Single question assessing the use of any tobacco- or nicotine-containing products in the last 6 months.

A positive score for anxiety (score at or above 10), depression (score at or above 10), cannabis/marijuana use (score at or above 1) or tobacco/nicotine use (score at or above 1) triggers step 2 (conditional screening) and subsequently step 3 (clinical assessment and treatment), while a positive score for sleep disturbance (score at or above 8), disordered eating (score at or above 15) or alcohol misuse (score at or above 8) triggers directly step 3 (clinical assessment and treatment). As in the IOC SMHAT-1, a positive answer given on item 9 of the PHQ-9 (ie, suicidal ideation) triggers direct action.

#### Step 2: conditional screening

A positive score in step 1 (universal screening) for symptoms of anxiety or depression triggers the completion of the Primary Care Post Traumatic Stress Disorder Screen for DSM-5 (PC-PTSD-5), assessing the presence of symptoms of PTSD in the past month, as the intersection of these symptoms is often seen in clinical practice.^35 36^ A positive score in step 1 (universal screening) for cannabis/marijuana use or tobacco/nicotine use triggers the completion of the Cutting Down, Annoyance by Criticism, Guilty Feeling and Eye-openers Adapted to Include Drugs (CAGE-AID), assessing the presence of substance misuse in the last 3 months (no focus on alcohol use as already explored with the AUDIT; additional question to explore which substance was used).^37–42^ Further information about these two existing validated screening questionnaires (including measurement properties) is presented as online supplemental material 5. Regardless of the score on the PC-PTSD-5 and CAGE-AID, the athlete proceeds to step 3 (clinical assessment).

#### Step 3: clinical assessment and treatment

Considering the previous outcomes (step 1 and/or step 2), a comprehensive clinical assessment is conducted by a sports medicine physician and/or licensed/registered mental health professional (eg, clinical psychologist, psychiatrist) in order to obtain additional relevant information (eg, mental health history, history of and/or presence of harassment/abuse within or outside of sports) and ultimately to identify either a clinical diagnosis or a scenario where brief intervention is required to support an athlete in a context-appropriate way as they are experiencing a transient, challenging situation. For such a clinical diagnosis (or for each of the identified diagnoses), especially severity, complexity, diagnostic uncertainty and previous treatment non-response are assessed (definition clearly presented in the IOC SMHAT2). If the sports medicine physician is uncomfortable determining the severity and/or complexity of a given athlete’s diagnosis, then referral to a licensed/registered mental health professional or to the most qualified healthcare provider available may be made. Based on all available information, the sports medicine physician and/or licensed/registered mental health professional chooses one of the following three actions:

- In cases that are neither severe, complex, diagnostically uncertain nor previously non-responsive to treatment, then treatment/support can be provided by a sports medicine or primary care physician, referring then to the *International Olympic Committee consensus statement on mental health in elite athletes (2026*) for clinical guidance for treatment.^4^
- In cases of diagnostic uncertainty or when further information might be useful, the sports medicine physician and/or licensed/registered mental health professional should consider whether one or more additional screening questionnaires should be completed by the athlete prior to making a definitive diagnosis. This includes, but is not limited to, screening for attention-deficit/hyperactivity disorder, bipolar disorder, gambling disorder, prolonged grief, obsessive-compulsive disorder and/or psychosis.^43–54^ The additional information gathered can be used to inform the diagnosis and creation of a treatment plan.
- In cases that are severe, complex, diagnostically uncertain (even after additional screening is completed) and/or non-responsive to treatment, we recommend referral of the athlete to a licensed/registered mental health professional (eg, clinical psychologist, psychiatrist) or to the most qualified healthcare provider available for further assessment and treatment.

#### Use of the IOC SMHAT2

When implementing the IOC SMHAT2, it is important for clinicians to understand the contextual nuances (eg, injury history, change in team, retirement) of the athlete’s reality to best interpret the results.^55^ The IOC SMHAT2 should be embedded within the pre-season period (ie, ideally a few weeks after the start of sport training but before the competition period). The IOC SMHAT2 can also be used mid- and end-season or when any significant event for athletes occurs, such as injury, illness, surgery, an unexplained performance concern, after a major competition, end of competitive cycle, suspected harassment/abuse, adverse life event and/or transitioning out of sport. Whether concerns have been raised or not, it is essential to provide athletes with feedback after completion of the IOC SMHAT2 to maintain compliance and trust as well as to improve mental health literacy.

The IOC SMHAT2 is available as a paper version (online supplemental file 4). In order to assist with the scoring of step 1 (universal screening) and step 2 (conditional screening) of the IOC SMHAT2, a calculator tool is also available as an Excel file. Both step 1 and step 2 of the IOC SMHAT2 could also be embedded in a medical-level privacy-secured online platform. The IOC SMHAT2 can be freely copied in its current form, without modifications, for distribution to individuals, teams, groups and organisations. Any modification requires approval from the IOC MHWG. To strengthen its multicultural use, we encourage language translation of the IOC SMHAT2 in collaboration with the IOC MHWG. The IOC SMHAT2 should not be re-branded or used for commercial gain.

## Discussion

Following the *International Olympic Committee consensus statement on mental health in elite athletes 2026*,^4^ and based on scientific and anecdotal reflections on its first version, we developed the IOC SMHAT2 to assess and manage mental health symptoms and disorders in elite athletes throughout their sporting career. Analogous to the IOC SMHAT-1, the IOC SMHAT2 is also comprised of a three-step approach, starting with a universal screening step (step 1), potentially followed by a secondary screening step (step 2) and a clinical assessment (step 3). However, significant differences between the first and second version of the tool lie in the composition of steps 1 and 2, in the mental health symptom clusters and related instruments selected for these two steps, and in the flow from step 1 to either step 2 or step 3. Regardless of these differences, it remains important to emphasise that *a positive score in the universal screening step (step 1) and/or conditional screening step (step 2) does not automatically provide a diagnosis for the given mental health symptom cluster but is a prompt for further clinical evaluation (step 3)*.

For step 1, the IOC MHWG decided to move away from a short triage step based on the 10 items of the APSQ and to start with a comprehensive screening step focusing on seven key mental health symptom clusters. The principal reason for this choice was that the scientific literature, the view of experienced users, and the analysis of the IOC MHWG data collected across sports, countries and cultures (all presented in previous sections) put forward notable concerns about the FNR and FPR of the APSQ (eg, up to 67% FNR for disordered eating). This concern has also been confirmed in a recent article indicating the APSQ’s lack of sensitivity to a broad range of mental health symptoms.^56^ Another reason was that the IOC MHWG believed the time investment required to complete a longer screening step as step 1 (estimated at 10 min) should not be a barrier in the context of elite sports. Indeed, elite athletes are regularly (eg, pre-season) screened for other medical conditions (eg, cardiovascular diseases) with instruments requiring as much or even more time. If the elite sports ecosystem puts the athletes’ mental health at the forefront of their medical support, then such a time investment should be negligible.

The seven key mental health symptom clusters included in the universal screening step (step 1) of the IOC SMHAT2 were critically assessed by the IOC MHWG, but those remained unchanged (being already explored in step 2 of the IOC SMHAT-1). In contrast, for the sake of consistency as well as due to their demonstrated utility, the IOC MHWG chose to use the same instruments (ie, GAD-7, PHQ-9, ASSQ); the instruments used to measure the other key mental health symptom clusters were changed. The most significant change was the removal of the BEDA-Q for the EDE-QS. The reason for this initial choice was that the BEDA-Q had been developed for and validated in an (elite) athlete population. However, the scientific literature, the view of experienced users, and the analysis of the IOC MHWG data (all presented in previous sections) confirmed the concern related to the BEDA-Q’s cut-off and its misclassifications. Consequently, the IOC MHWG selected the EDE-QS to assess the presence of disordered eating because it is comprised of a limited number of items (ie, 12) and it has robust measurement properties (eg, area under receiver operating characteristic curve: 0.85–0.95). Another change was to replace the 3-item AUDIT-C with the 10-item AUDIT. The IOC MHWG believed that the use of the longer version was justified given the substantial prevalence of alcohol misuse among elite athletes (eg, around 50% in Dutch Olympic/Paralympic athletes), while it would potentially provide relevant additional information for the clinical assessment (step 3).^2 6 8^ A last noteworthy change was to explore the use of cannabis/marijuana- and tobacco/nicotine-containing products in the last 6 months with two single questions (yes or no). Because an athlete either had or had not used these products in the last 6 months, the IOC MHWG determined that these two single questions were sufficient to potentially trigger the completion of the CAGE-AID in step 2, assessing for the presence of substance misuse.

The conditional screening step (step 2) of the IOC SMHAT2 includes only two key mental health symptom clusters. The presence of substance misuse is assessed with the CAGE-AID as it already was in the IOC SMHAT-1. The presence of PTSD was also measured with the PC-PTSD-5 in the IOC SMHAT-1, but only as optional screening following the clinical assessment. In the IOC SMHAT2, this key mental health symptom cluster is moved to the forefront. This change resulted from the IOC MHWG’s reflections as well as the important view of experienced users acknowledging the clinical relevance of PTSD in athletes.

The flow from step 1 to either step 2 or step 3 was revised, in parallel with the redefinition of the universal and conditional screening steps (step 1 and step 2). A positive score for anxiety (GAD-7) and/or depression (PHQ-9) triggers the conditional screening step (step 2) and the completion of the PC-PTSD-5 as PTSD, anxiety and depression often intersect and often relate to exposure to a traumatic event (either inside or outside sport).^57–59^ A positive score for cannabis/marijuana use or tobacco/nicotine use triggers the conditional screening step (step 2) and the completion of the CAGE-AID to explore for the presence of substance misuse. Regardless of the score on the PC-PTSD-5 or CAGE-AID, a clinical assessment (step 3) always follows the conditional screening step (step 2). This progression pathway differs from the IOC SMHAT-1. A second noteworthy difference in the flow of the IOC SMHAT2 is that a positive score on either the ASSQ (sleep disturbance), EDE-QS (disordered eating) or AUDIT (alcohol misuse) directly triggers the clinical assessment (step 3). Such a choice was made because of the substantial prevalence of these three key mental health symptom clusters among elite athletes as well as their potential impact on performance and quality of life.^2^

Step 3 (clinical assessment and treatment) of the IOC SMHAT2 remained mostly unchanged. The authors would like to emphasise that the sports medicine physician and/or licensed/registered mental health professional conducting the clinical assessment ideally should already have developed a therapeutic trusting relationship with the athlete so that (1) the athlete’s symptoms can be evaluated with respect to their cultural context and (2) the athlete may be more receptive to feedback from the assessor, which may help to build compliance and mental health literacy.

The IOC SMHAT2 is expected to be a step forward as a screening tool to identify elite athletes potentially experiencing mental health symptoms and disorders, concurring with the sustainable commitment of the IOC to provide the elite sports ecosystem with mental health support and resources. While such an expectation would also align with the most recent findings among Team USA athletes that support the ongoing use of mental health screening, the scientific and clinical added value of the IOC SMHAT2 will need to be established in the upcoming years.^60^ As for its first version counterpart, the IOC SMHAT2 presents some limitations, and its implementation is likely to face various challenges.

First, the information gathered via literature (stage 1 and 3), semi-structured interviews (stage 2) and surveys (stage 6) was collected in English. This linguistic restriction might have limited participation and insight from other regions such as Africa and Latin America, as well as the representativeness of the findings. Furthermore, as 3 out of 14 studies were captured for our scoping reviews (stages 1 and 3) from the reference lists, one might argue that our search strategy was inadequate. Even more, our scoping review conducted for stage 3 was directed towards quantitative studies while information from qualitative studies (if any) might have been informative as well, and our validation (stage 6) was conducted among intended end users (healthcare professionals) but not among patients/athletes.

Second, the semi-structured interviews (stage 2) were conducted remotely, which might have reduced the establishment of rapport or access to non-verbal cues. However, the authors did not perceive any constraints linked to remote video interviewing.

Third, the validated screening questionnaires selected for the IOC SMHAT2 are directed towards mental health symptoms, but these do not assess well-being. This might be detrimental to the assessment of the full continuum of mental health. Also, these validated screening questionnaires provide self-reported information subject to bias, while some are not developed for and validated specifically in elite athlete populations. Furthermore, these validated screening questionnaires do not reach 100% sensitivity and 100% specificity, implying the potential for misclassification. Therefore, future validation studies should be conducted among elite athletes from various sport disciplines and cultures, while the development of other screening approaches and tools should be considered (as recently presented in Poland).^61^ Even more, combining screening based on self-report with brief clinical interviews by qualified health professionals (eg, sports medicine, sport psychologists) with clinical training was recently suggested to mitigate the potential for misclassification, but this approach may not be feasible in under-resourced sports and countries.^62^

Fourth, mental health remains stigmatised among the general population and athletic population in many countries and regions. This might impair the use of the tool as part of a regular screening programme in parallel with other athlete screening (eg, musculoskeletal and cardiovascular screening).

Fifth, while its first version is available in nine languages (eg, French, German, Persian, Spanish), the IOC SMHAT2 is only available in English at the present time. This will form a barrier to its use; however, as for the IOC SMHAT-1, the IOC MHWG anticipates that various research groups will be interested in translating the tool into their respective languages as well as in culturally adapting the tool.

Sixth, the availability of sports medicine physicians and other licensed/registered health professionals, as well as licensed/registered mental health professionals specifically, cannot be taken for granted in many countries. This is even more of a concern in developing countries. This reality implies that an athlete might not be referred to a mental health professional but rather to the most qualified support provider available. In that case, the IOC Sport Mental Health Recognition Tool 2 (IOC SMHRT2) can be used by athletes, coaches, family members and all other members of the athlete’s entourage as an awareness tool to facilitate early detection of mental health symptoms and promote help-seeking behaviour (online supplemental material 6).

Seventh, the time needed to use the IOC SMHAT2 ranges from 10 min for step 1 only to 60 min for step 1, step 2 and step 3 (the number of questions in steps 1 and 2 of the IOC SMHAT2 is slightly more than in the IOC SMHAT-1, ie, 54 vs 47). Although this could be considered an additional challenge to its use, prioritising mental health negates this concern.

Finally, the implementation of the IOC SMHAT2 can only be successful if the elite sports ecosystem prioritises addressing the aforementioned challenges and is characterised by a leadership mission and vision that foster a positive (sport) culture and prioritise athlete performance, health and quality of life.

## Conclusion

Following the *International Olympic Committee consensus statement on mental health in elite athletes 2026* and based on scientific and anecdotal reflections on its first version, we developed the IOC SMHAT2 as a second, updated version of the tool to identify elite athletes potentially experiencing mental health symptoms and disorders. Aligning with the sustained commitment of the IOC to improve the mental health of elite athletes, the IOC SMHAT2 should facilitate timely referral of those in need of support and/or treatment. Future validation studies should be continued among elite athletes from various sports disciplines and cultures. Ultimately, the goal of this project is to improve the identification of elite athletes who are experiencing mental health symptoms and disorders to maximise their health and well-being as well as to assist them in achieving their sport and life goals.

## Supplementary material

10.1136/bjsports-2025-111408 online supplemental file 1

10.1136/bjsports-2025-111408 online supplemental file 2

10.1136/bjsports-2025-111408 online supplemental file 3

10.1136/bjsports-2025-111408 online supplemental file 4

10.1136/bjsports-2025-111408 online supplemental file 5

10.1136/bjsports-2025-111408 online supplemental file 6

## Data Availability

All data relevant to the study are included in the article or uploaded as supplementary information.
