# Supplementary material for: International Olympic Committee Sport Mental Health Assessment Tool 2 (IOC SMHAT2): development, content validity and feasibility
Source: Br J Sports Med. 2026 Jul 16;60(15):e111408. doi: 10.1136/bjsports-2025-111408 (PMC13479680; doi:10.1136/bjsports-2025-111408)
Supplement: Supplementary file 1 [file bjsports-60-15-s002.pdf]

Online supplementary material 2

Studies involving the International Olympic Committee (IOC) Sport Mental Health Assessment Tool 1 (SMHAT-1)

Study information	Study population	Study design	Strengths and limitations	Misclassification and measurement properties
Anderson et al. (2023)[8]	N: 1,066 athletes<br>G: Men: 48.6% and Women: 51.4%<br>A: Not specified<br>S: 51 different Olympic and Paralympic summer and winter sports<br>L: Olympic: 90.9% Paralympic: 9.1%<br>C: United States	Cross-sectional study<br><br>Response rate: 100% as all Team USA athletes were required to complete the SMHAT-1 as part of their health history screening for the Tokyo 2020 and Beijing 2022 Games.<br><br>Follow-up: All athletes screened positive on any of the SMHAT-1 questionnaires received a clinical follow-up within 48 hours.	Strengths:<br>It is a standardised mental health screening tool. It is in its infancy state which means it is expected to be modified and changed based on data collected.<br><br>Limitations:<br>There is a wide range of false negative rates (4.8%–66.7%) using the APSQ, - meaning many athletes with mental health concerns would not be identified if only the APSQ was used as a screening tool. APSQ should not be used as a stand-alone initial triage step in the use of the SMHAT-1 in athletes.	False negative of step 1 (versus step 2)<br>Anxiety: 5%<br>Depression: 5%<br>Suicidal ideation: 7%<br>Sleep disturbance:39%<br>Alcohol misuse: 60%<br>Drug misuse: 46%<br>Disordered eating: 67%<br><br>False positive of step 1 (versus step 2)<br>Anxiety: 27%<br>Depression: 28%<br>Suicidal ideation: 29%<br>Sleep disturbance: 23%<br>Alcohol misuse: 24%<br>Drug misuse: 68%<br>Disordered eating: 3%
Bilgoe et al. (2024)[7]	N: 156 athletes<br>G: Men 45%, Women 55%<br>A: 26.1 ± 7.5<br>S: Olympic and Paralympic sports<br>L: Elite<br>C: Netherlands	Cross-sectional study<br><br>Response rate: Not reported exclusively for athletes.<br><br>Follow-up: None	Strengths:<br>SMHAT-1 was implemented at a single time point to detect mental health symptoms among Dutch elite athletes, thereby increasing awareness of mental health symptoms among these athletes.<br><br>Limitations:<br>SMHAT-1 is an online questionnaire in which mental health symptoms are self-reported and not clinically diagnosed by a professional. This may introduce subjectivity into the results and potentially lead to either an underestimation or overestimation of the problem's extent.	Misclassification not explored<br><br>Measurement properties not explored
Bilgoe et al. (2024)[12]	N: 74 athletes<br>G: Women 100%<br>A: 25.0 ± 2.7<br>S: Football<br>L: Professional	Cross-sectional study with repeated measurements	Strengths:<br>SMHAT-1 covers a wide range of mental health symptoms (*e.g.*, distress, anxiety, sleep disturbances). The questionnaire allows early	Misclassification not explored<br><br>Measurement properties not explored

	C: Various	Response rate: Not reported<br><br>Follow-up: T1 (6 months postbaseline), T2 (12 months postbaseline)	detection and raises mental health awareness among elite athletes.<br><br>Limitations:<br>SMHAT-1 is a validated tool for assessing mental health symptoms at a single time point; however, this study is longitudinal. Additionally, as the questionnaire relies on self-reporting, it may introduce subjectivity into the results.	
Kilic et al. (20241)[5]	N: 281 active football players, 81 retired football players<br>G: Men 53% Women 47%<br>A: Active players men 24.3 ± 4.8, Active players women 22.8 ± 4.0, Retired players 38.7 ± 6.0<br>S: Football<br>L: Professional<br>C: Australia	Cross-sectional study<br><br>Response rate: Active players 62%, Retired players 22%<br><br>Follow-up: None	Strengths:<br>SMHAT-1 helps to identify athletes in need of mental health treatment. SMHAT-1 includes validated instruments and is part of the IOC mental health toolkit.<br>SMHAT-1 covers multiple mental health domains and should be used to set up a multidisciplinary approach in tackling mental health symptoms of football players.<br><br>Limitations:<br>Measurement of mental health symptoms through self-reporting (questionnaires) might lead to recall bias and underreporting.	Misclassification not explored<br><br>Measurement properties not explored
Mountjoy et al. (2023)[6]	N: 542<br>G: Men 54%, Women 46%<br>A: 21.0 ± 1.7 years<br>S: 17 university sports (Team: 369, Individual: 174)<br>L: Elite university-level athletes (varsity athletes)<br>C: Canada	Cross-sectional study with repeated measurements<br><br>Response rate: 98% at T0 (October 2020), 61% at T1 (January 2021), 24% at T2 (April 2021)<br><br>Follow-up: Medical care provided as needed. The process followed SMHAT-1's clinical triage steps 3a/3b for positive screens.	Strengths:<br>SMHAT-1 tool was successfully implemented 3 times across an academic year.<br>Captures mental health symptoms across distress, anxiety, depression, sleep, substance use, eating disorders.<br>Implementing the SMHAT-1 successfully collected information on the mental health status of student-athletes throughout the academic year (this should increase detection of mental health symptoms in university-level athletes)<br><br>Limitations:<br>SMHAT-1 is only validated as a single measurement tool, this study applied it longitudinally. SMHAT-1 is an online self-reporting tool that may lead to the underreporting of symptoms.	Misclassification not explored<br><br>Internal consistency<br>APSQ: α = 0.84<br>GAD-7: α = 0.90<br>PHQ-9: α = 0.87<br>AUDIT-C: α = 0.81<br>BEDA-Q: α =0.70<br>ASSQ: α = 0.66<br>CAGE-AID: α =0.69
Ojio et al.	N: 220	Cross-sectional study	Strengths:	False negative of step 1 (versus step 2)

(2023)[20]	G: Men 100%<br>A: 28.0 years (SD = 3.98)<br>S: Professional rugby (Japan Rugby Top League)<br>L: Elite/ professional national-level<br>C: Japan	Response rate: 40.6%<br><br>Follow-up: None<br>No detailed mention of clinical follow-up or care pathways initiated from the screening	Step 1 of the SMHAT-1 successfully detected all positive cases for anxiety, depression, and suicidal ideation. Low false-negative rates (FNR) for sleep disturbance (9.1%) and substance misuse (2.6%)<br><br>Limitations:<br>APSQ gives high false-negative rates for alcohol misuse (64,9%) and eating disorders (35.1%).<br>Further refinement of the SMHAT-1 is needed to reduce false negatives.<br>Several screening tools are used and translated but not validated to the Japanese populations.	Anxiety: 0%<br>Depression: 0%<br>Suicidal ideation: 0%<br>Sleep disturbance: 9%<br>Alcohol misuse: 65%<br>Drug misuse: 3%<br>Disordered eating: 35%
Pillay et al. (2024)[11]	N: 101<br>G: Men 100%<br>A: 26.5 years (SD = 1.7)<br>S: Professional footballers<br>L: Elite / professional<br>C: European countries	Cross-sectional study with repeated measurements<br><br>Response rate to follow up questionnaire: 79.2%<br><br>Follow-up: 12-months after baseline	Strengths:<br>None mentioned.<br><br>Limitations:<br>All screening tools relied on self-reporting of symptoms, and therefore, under- or overreporting may have occurred.	Misclassification not explored<br><br>Measurement properties not explored
Saifi et al. (2024)[21]	N: 25<br>G: Women 100%<br>A: 20.5 ± 2.38<br>S: Football<br>L: State-level<br>C: India	Cross-sectional study<br><br>Response rate: 100%<br><br>Follow-up: No mention of follow-up	Strengths:<br>The SMHAT-1 is mentioned as a tool used to assess the mental health status of the participants, but the study does not explicitly outline the specific benefits of using the SMHAT-1 in the context of this research.<br>The study refers to the SMHAT-1 being validated by the International Olympic Committee (IOC) and notes that it was useful for screening mental health issues in the participants.<br><br>Limitations:<br>None mentioned.	Misclassification not explored<br><br>Measurement properties not explored
Waleriańczyk et al. (2024)[22]	N: 1,121<br>G: Men 51.4%, Women 48.6%<br>A: 24.4 ± 5.1<br>S: Various Olympic sports	Cross-sectional study<br><br>Response rate: 96%<br><br>Follow-up: SMHAT-1 procedure is followed by	Strengths:<br>SMHAT-1 provides a useful extension of routine medical check-ups for elite athletes.<br><br>Limitations:	False negative of step 1 (versus step 2)<br>Any construct: 18%<br>Anxiety: 3%<br>Depression: 1%<br>Suicidal ideation: 3%<br>Sleep disturbance: 16%

	L: Olympic athletes<br>C: Poland	a brief 10-15 min interview with a qualified sports psychologist. Types of clinical follow up included: no action, psychoeducation or consultation sports psychologist, referral to psychotherapist or psychiatrist.	SMHAT-1 does not have indicators of well-being only of ill-being. SMHAT-1 uses measures with questionable reliability and arbitrary cut-off points.<br>Triage cut-off of ≥ 17 was associated with a very high negative predictive value and low positive predictive value, a threshold of ≥23 would provide optimal sensitivity and specificity.<br>Using only the SMHAT-1 scores, only 49% of athletes would be classified as having good mental health, with the additional interview no mental health action is required in 68.2%.<br>Additional interviews elevated the efficacy of mental health evaluation.	Alcohol misuse: 18%<br>Drug misuse: 6%<br>Disordered eating: 17%<br><br>False positive at step 1 (versus step 2)<br>Any construct: 62%<br>Anxiety: 71%<br>Depression: 70%<br>Suicidal ideation: 72%<br>Sleep disturbance: 69%<br>Alcohol misuse: 70%<br>Drug misuse: 71%<br>Disordered eating: 70%<br><br>False positive at step 1 (versus step 3)<br>72% versus 10%<br><br>False positive at step 1 (versus step 3)<br>51% versus 10%
Wheelan et al. (2025)[23]	N: 2,758<br>G: Men 52%, Women 48% (based on 742 participants as 2,016 did not report sex assigned at birth)<br>A: Median 20 (range 17 – 26)<br>S: All NCAA division I sports<br>L: Collegiate elite level (NCAA division I)	Cross-sectional study<br><br>Response rate: Not reported<br><br>Follow-up: No long-term follow-up as study used cross-sectional data collected between June 2020 and June 2023 (30% of athletes identified via APSQ as needing additional mental health screening)	Strengths:<br>The study examined the use of APSQ within the SMHAT-1 framework, which can be quickly completed which is practical for time-limited settings. The APSQ has a high accuracy in detecting anxiety and depression.<br><br>Limitations:<br>There are high false-negative rates for sleep disorders, alcohol and substance misuse, and eating disorders.<br>APSQ lacks any item assessing suicidality, adding a single suicide-risk question could substantially improve the clinical value of the screening tool without lowering compliance.<br>Using BEDA-Q as an additional screening tool for identifying eating disorders is suggested in the SMHAT-1, however this screening tool has a poor specificity, the EDE-QS has shown to have a stronger reliability where the false negative rate was reduced from 66.7% (in a study of Olympic and Paralympic athletes) to 18% (this study).	False negative of step 1 (versus step 2)<br>Anxiety: 8%<br>Depression: 13%<br>Suicidal ideation: 9%<br>Sleep disturbance: 44%<br>Alcohol misuse: 53%<br>Drug misuse: 37%<br><br>False positive at step 1 (versus step 2)<br>Anxiety: 24%<br>Depression: 23%<br>Suicidal ideation: 26%<br>Sleep disturbance: 23%<br>Alcohol misuse: 26%<br>Drug misuse: 29%

	Adding specific questions on sleep, substance use, eating disorders and suicidality to the APSQ could improve accuracy and efficiency.<br>Keeping a limited number of questions asked to 15-20 helps to decrease time burden and increase compliance.<br>APSQ should be tested across different contexts (various sports, divisions, cultural and socioeconomic backgrounds) to improve generalizability.	

N, number of participants; G, gender; A, mean age (years); S, type of sport; L, level of competition; C, country
