# Supplementary material for: International Olympic Committee Sport Mental Health Assessment Tool 2 (IOC SMHAT2): development, content validity and feasibility
Source: Br J Sports Med. 2026 Jul 16;60(15):e111408. doi: 10.1136/bjsports-2025-111408 (PMC13479680; doi:10.1136/bjsports-2025-111408)
Supplement: Supplementary file 2 [file bjsports-60-15-s004.pdf]

International
Olympic
Committee

Developed by the
IOC Mental Health
Working Group

IOC SMHAT2
THE INTERNATIONAL OLYMPIC COMMITTEE SPORT MENTAL HEALTH ASSESSMENT TOOL 2

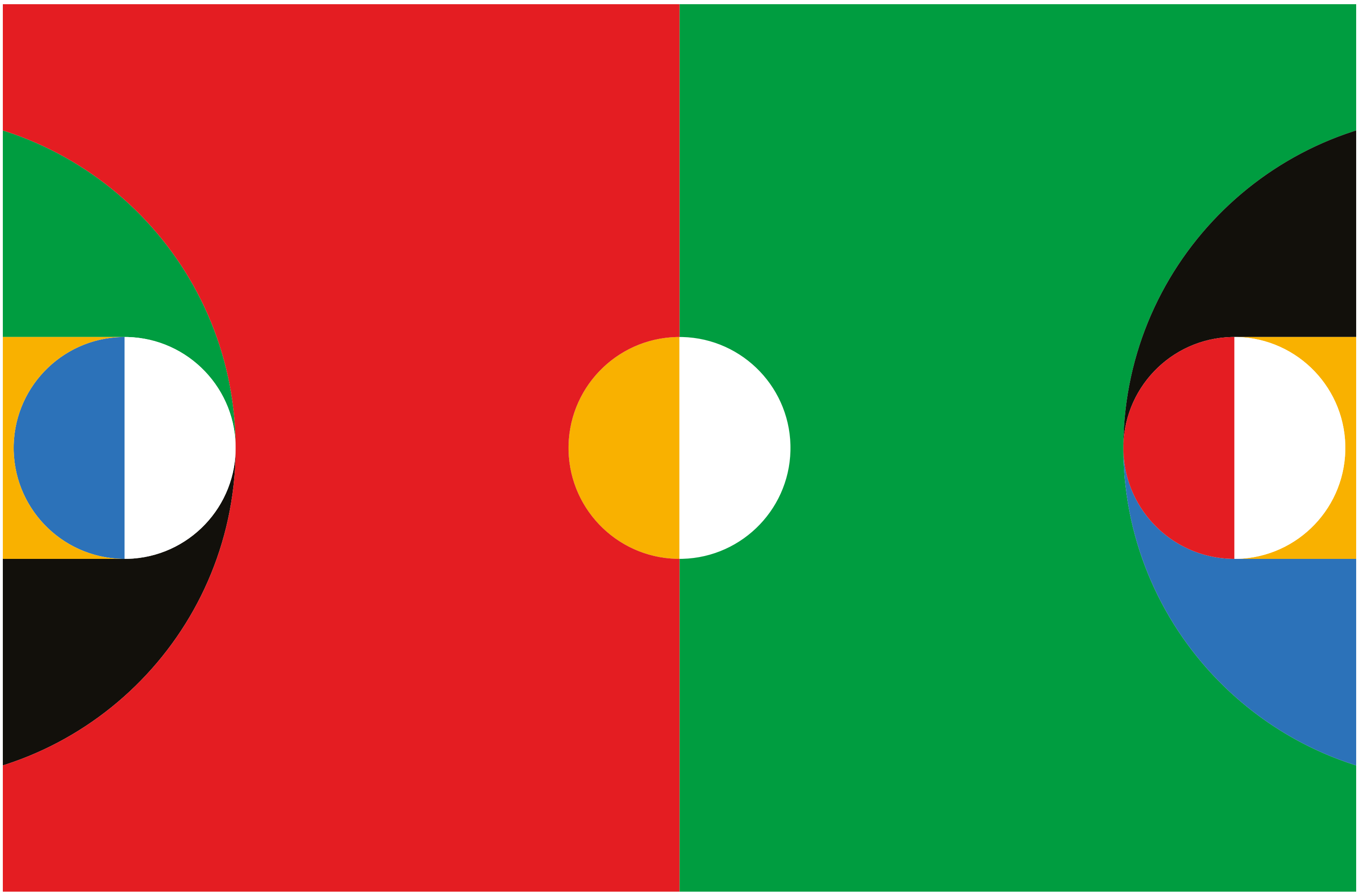

IOC SMHAT2

The International Olympic Committee Sport Mental Health Assessment Tool 2
Developed by the IOC Mental Health Working Group

International Olympic Committee

ATHLETE'S NAME: ....

ATHLETE'S ID NUMBER: ....

DATE: ....

MEDICAL FORM

What is the IOC SMHAT2

The International Olympic Committee (IOC) Sport Mental Health Assessment Tool 2 (SMHAT2) is a standardised assessment tool aiming to identify elite athletes (defined as professional, Olympic or collegiate level; 16 years of age and older) potentially experiencing mental health symptoms and/or disorders, to facilitate timely referral of those in need of support and/or treatment. Developed by the IOC Mental Health Working Group (MHWG), the IOC SMHAT2 relies on a three-step approach including universal screening as Step 1 presented in the Athlete form 1 (10 minutes to complete), conditional screening as Step 2 presented in the Athlete form 2 (5 minutes to complete), and an aid to clinical assessment as Step 3 presented in the Medical form (30-45 minutes to complete).

Who should use the IOC SMHAT2

The IOC SMHAT2 can be used by sports medicine physicians and other licensed/registered health professionals, but the clinical assessment (and related management) within the IOC SMHAT2 (see Step 3) should be conducted by sports medicine physicians and/or licensed/registered mental health professionals. If you are not a sports medicine physician or other licensed/registered health professional, please use the IOC Sport Mental Health Recognition Tool 2. Physical therapists or athletic trainers working with a sports medicine physician can use the IOC SMHAT2, but the clinical assessment, guidance and/or intervention should remain the responsibility of a sports medicine physician or a licensed/registered mental health professional.

Why use the IOC SMHAT2

Mental health symptoms and disorders are prevalent among active and former elite athletes. Mental health disorders are syndromes characterised by a clinically significant disturbance in an individual's cognition, emotional regulation or behaviour that reflects a dysfunction in the psychological, biological and/or developmental processes that underpin mental and behavioural functioning. These disturbances are associated with distress or impairment in personal, family, social, educational, occupational or other important areas of functioning and are diagnosed according to existing clinical criteria such as in the Diagnostic and Statistical Manual of Mental Disorders Fifth Edition Text Revision (DSM-5-TR) or the International Classification of Diseases, 11th Revision (ICD-11). Mental health symptoms are any self-reported adverse thought, feeling, behaviour and/or psychosomatic symptom that might lead to subjective distress or functional impairments in daily life, work and/or sport.

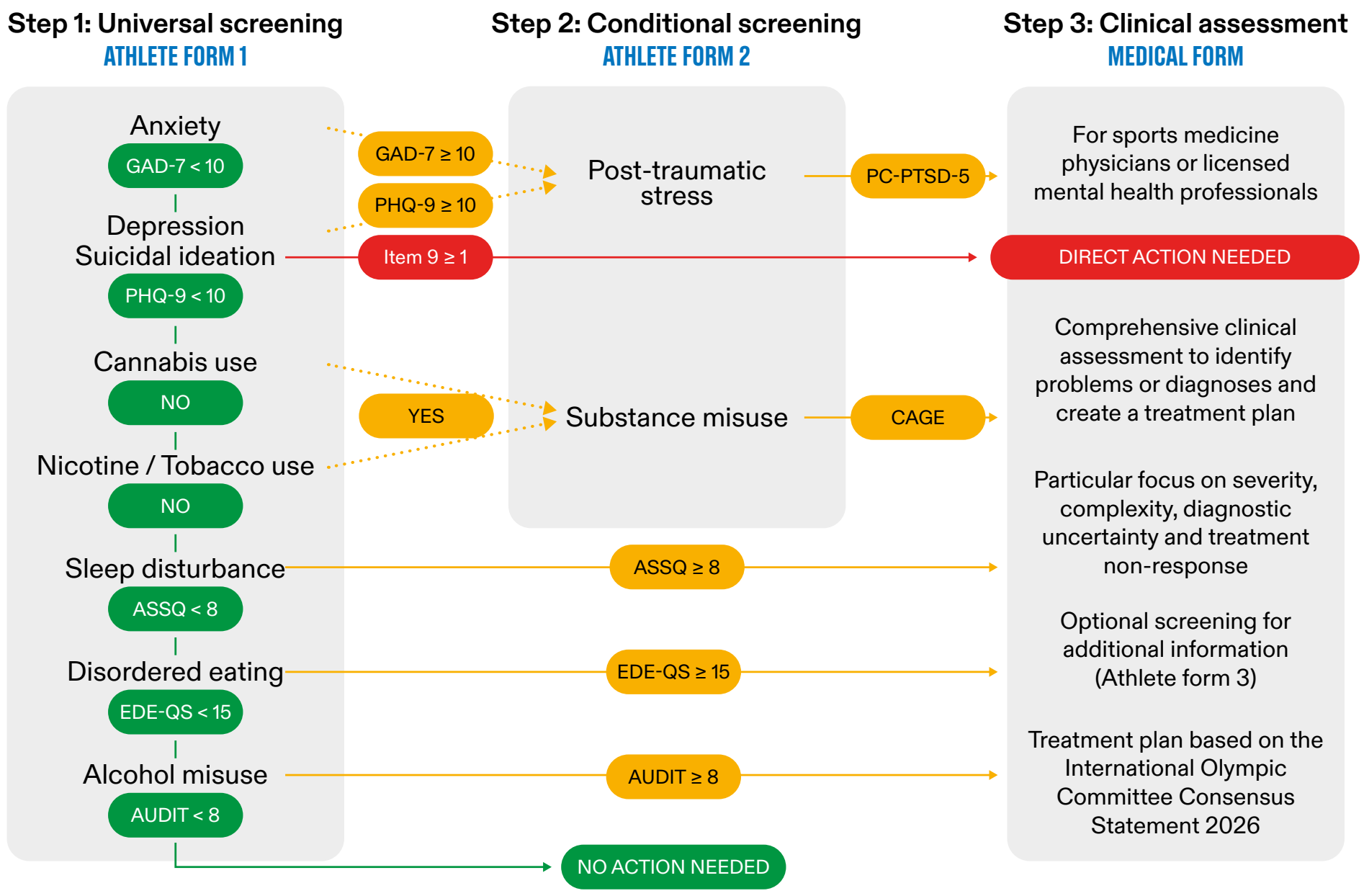

When to use the IOC SMHAT2

The IOC SMHAT2 should be embedded within the pre-season period (i.e., ideally a few weeks after the start of sport training but before the competition period).

The IOC SMHAT2 can also be used mid- and end-season or when any significant event for athletes occurs such as injury, illness, surgery, any significant performance concern, after a major competition, end of competitive cycle, suspected harassment/abuse, adverse life event, deselection and/or transitioning out of sport.

The IOC SMHAT2 and its related screening instruments should be used with strict attention to confidentiality and data security to ensure privacy, trust and psychological safety (e.g., that the results will not be shared without the participant's consent and not used for selection processes).

IOC SMHAT2 calculator tool

In order to assist with the scoring of Step 1 (universal screening) and Step 2 (conditional screening) of the IOC SMHAT2, you can use the IOC SMHAT2 calculator tool (Excel file to download via QR code). In this Excel file, two input sheets for the athlete are visible, while two calculation sheets and one medical form sheet for the sports medicine physician or licensed/registered mental health professional are hidden.

The first input sheet is related to universal screening (Step 1; Athlete form 1) while the second input sheet is related to conditional screening (Step 2; Athlete form 2). Both input sheets can be completed by the athlete (Athlete form 2 only if applicable). To access the two calculation sheets with all total scores as well as the medical form sheet, right-click on any sheet tab at the bottom of the file, click "Unhide...", select all three sheets you want to unhide in the Unhide window, and click OK. If you prefer to use the paper version of the IOC SMHAT2, please print it single-sided.

SCAN THE QR CODE TO DOWNLOAD THE IOC SMHAT2 CALCULATOR TOOL

The IOC SMHAT2 in its current form can be freely copied for distribution to individuals, teams, groupsand organisations. Any modification requires the specific approval by the IOC MHWG, while any translation should be reported to the IOC MHWG. The IOC SMHAT2 must not be re-branded or sold for commercial gain. Further information about the development of the IOC SMHAT2 and related screening tools (including psychometric properties) is presented in the corresponding publication of the British Journal of Sports Medicine.

STEP 1: UNIVERSAL SCREENING FOR MENTAL HEALTH SYMPTOMS AND DISORDERS

1

Action Have the athlete complete the screening 1-7 in the Athlete form 1 exploring 7 key mental health symptom clusters via 7 questionnaires (in total 45 items).

Action Calculate the total score for each key mental health symptom cluster and related questionnaire by summing up the athlete responses. Note the scores in the following table and tick the appropriate box(es).

	Total score	Under threshold		At or above threshold		Action
Anxiety (screening 1)		0-9	☐	≥10	☐	Step 2 and Step 3
Depression (screening 2)		0-9	☐	≥10	☐	Step 2 and Step 3
Depression item 9 (screening 2)		0	☐	≥1	☐	ACTION NEEDED
Cannabis use (screening 3)		0	☐	1	☐	Step 2 and Step 3
Nicotine/Tobacco use (screening 4)		0	☐	1	☐	Step 2 and Step 3
Sleep disturbance (screening 5)		0-7	☐	≥8	☐	Step 3
Disordered eating (screening 6)		0-14	☐	≥15	☐	Step 3
Alcohol misuse (screening 7)		0-7	☐	≥8	☐	Step 3

Anxiety:
score 5-9 = mild;
score 10-14 = moderate;
score ≥15 = severe

Depression:
score 5-9 = mild;
score 10-14 = moderate;
score 15-19 = moderate/severe;
score ≥20= severe

Sleep disturbance:
score 5-7 = mild;
score 8-10 = moderate;
score ≥11 = severe

Alcohol misuse:
score 1-7 = low-risk consumption;
score 8-14 = hazardous or harmful consumption;
score ≥15 = moderate-severe alcohol use disorder

Box ticked for depression item 9 (screening 2)	>	Take immediate action (according to mental health emergency plan)
All screening scores under threshold	>	No action needed
Screening scores for Anxiety (screening 1) and/or Depression (screening 2) at or above threshold	>	Proceed to Step 2 (conditional screening) and subsequently to Step 3 (clinical assessment)
Screening scores for Cannabis use (screening 3) and/or Nicotine/Tobacco use (screening 4) at or above threshold	>	Proceed to Step 2 (conditional screening) and subsequently to Step 3 (clinical assessment)
Screening scores for Sleep disturbance (screening 5), Disordered eating (screening 6) and/or Alcohol misuse (screening 7) at or above threshold	>	Proceed to Step 3 (clinical assessment)

STEP 2: CONDITIONAL SCREENING TOOLS FOR MENTAL HEALTH SYMPTOMS AND DISORDERS

2

Action If screening scores for Anxiety (screening 1) and/or Depression (screening 2) are at or above threshold, have the athlete complete screening 8 (Post-traumatic stress) in the Athlete form 2.

Action If screening scores for Cannabis use (screening 3) and/or Nicotine/Tobacco use (screening 4) are at or above threshold, have the athlete complete screening 9 (Substance misuse) in the Athlete form 2.

Action Calculate the total score for each mental health symptoms cluster and related questionnaire by summing up the answers of the items. Note the scores in the following table and tick the appropriate box(es).

	Total score	Under threshold		At or above threshold		Action
Post-traumatic stress (screening 8)		0-2	☐	≥3	☐	Step 3
Substance misuse (screening 9)		0-1	☐	≥2	☐	Step 3

Regardless of the screening scores for Post-traumatic stress (screening 8) or Substance misuse (screening 9)	>	Proceed to Step 3 (clinical assessment)

3

STEP 3: CLINICAL ASSESSMENT AND TREATMENT

This step must be completed by a sports medicine physician or a licensed/registered mental health professional. The objective of this step is to conduct a comprehensive clinical assessment in order to evaluate the athlete's mental health, diagnose a disorder (if applicable), identify important contributing factors and create a relevant treatment plan. During this comprehensive clinical assessment, particular focus on severity, complexity, diagnostic uncertainty, and treatment response is recommended. Evaluation of the athlete's past medical/psychiatric history (if available) can be informative.

Action Read the following.

Severity

Severity refers to an identified clinical problem significantly compromising the athlete's health, safety or wellbeing. This can mean severe functional impairments, markedly disturbed behaviours and/or risk to self or others (e.g., suicidal/homicidal intent, significant self-neglect or electrolyte abnormalities in eating disorders would be considered high severity).

Complexity

Complexity refers to comorbid mental health and other medical conditions in which multiple conditions interact to make it challenging to diagnose and/or treat. Complexity might also include the interaction of significant sport (e.g., performance concerns, career dissatisfaction) or non-sport (e.g., relationship or financial problems, bereavement) stressors.

Diagnostic uncertainty

Diagnostic uncertainty refers to any doubt about diagnosis. Examples include differentiating a high level of sports related physical activity from over-activity found in hypomania and ADHD, functional performance-related eating from eating disorders, or depression from bipolar disorder.

Treatment non-response

Treatment non-response refers to one or more treatment cycles with no response or a partial response.

Action Review and interpret the screening scores (Step 1 and/or Step 2) and conduct a clinical assessment in order to obtain additional information. Inquire about a present or past history of harassment/abuse (interpersonal violence) within or outside of sport.

Action Complete the following table by ticking the appropriate box(es) and note your treatment recommendations for all relevant mental health symptoms clusters.

Problem	Severity	Complexity	Diagnostic uncertainty	Treatment non-response	Treatment recommendations
Anxiety	☐	☐	☐	☐	
Depression	☐	☐	☐	☐	
Sleep disturbance	☐	☐	☐	☐	
Disordered eating	☐	☐	☐	☐	
Alcohol misuse	☐	☐	☐	☐	
Post-traumatic stress	☐	☐	☐	☐	
Substance misuse	☐	☐	☐	☐	
	☐	☐	☐	☐	
	☐	☐	☐	☐	

In cases that are neither severe, complex, diagnostically uncertain nor non-responsive to treatment	>	Treatment/support can be provided by a sports medicine/primary care physician, referring then to the International Olympic Committee consensus statement on mental health in elite athletes (2026) for clinical guidance for treatment.
In cases of diagnostic uncertainty or when further information might be useful	>	Consider whether one or more additional screening tools should be completed by the athlete.<br>If relevant, use the Athlete form 3: screening 10 for Attention-deficit/hyperactivity disorder, screening 11 for Bipolar disorder, screening 12 for Gambling, screening 13 for Prolonged grief, screening 14 for Obsessive-compulsive disorder and screening 15 for Psychosis.<br>For the calculation of total score(s) and related interpretation of the Athlete form 3, please refer to the last section of this medical form.
In cases that are severe, complex, diagnostically uncertain even after any appropriate additional screening and/or non-responsive to treatment	>	Athletes should be referred to a licensed/registered mental health professional (e.g., clinical psychologist or psychiatrist) or to the most qualified support provider available.

Optional screening

3

Action According to your clinical assessment, consider having the athlete complete the relevant screening(s) in the Athlete form 3.

Action Calculate the total score for the relevant screening(s) by summing up the answers of the items and complete the following (if applicable).

Screening 10: Attention-deficit/hyperactivity disorder

	Total score
Calculate the total score by summing up the answers on the 6 items	

score ≥4 = symptoms highly consistent with ADHD

Screening 11: Bipolar disorder

	Total score
Calculate the total score by summing up the answers on item 1	
Note the score of item 2	
Note the score of item 3	

possible bipolar disorder if total score ≥7 AND item 2 = 1 AND item 3 = 1

Screening 12: Gambling

	Total score
Calculate the total score by summing up the answers on the 9 items	

score 0 = non-problem gambling;
score 1-2 = low level of problems with few or no identified negative consequences;
score 3-7 = moderate level of problems leading to some negative consequences;
score ≥8 = problem gambling with negative consequences and a possible loss of control

Screening 13: Prolonged grief

	Total score
Calculate the total score by summing up the answers on the 10 symptom items	

score ≥30 = prolonged grief disorder

Screening 14: Obsessive-compulsive disorder

	Total score
Calculate the total score by summing up the answers on the 18 items	

score ≥21 = likely presence of obsessive-compulsive disorder

Screening 15: Psychosis

	Total score
Calculate the total score by summing up the answers on the 16 items	

score ≥6 = at risk for psychosis

ATHLETE'S NAME: ....

ATHLETE'S ID NUMBER: ....

DATE: ....

ATHLETE FORM 1

Screening 1

The following questions relate to feeling anxious or stressed. Over the last 2 weeks, how often have you been bothered by the following problems? Please circle the answer that best represents how you have been.

	Not at all	Several days	More than half the days	Nearly every day
1. Feeling nervous, anxious, or on edge	0	1	2	3
2. Not being able to stop or control worrying	0	1	2	3
3. Worrying too much about different things	0	1	2	3
4. Trouble relaxing	0	1	2	3
5. Being so restless that it's hard to sit still	0	1	2	3
6. Becoming easily annoyed or irritable	0	1	2	3
7. Feeling afraid as if something awful might happen	0	1	2	3

Screening 2

The following questions relate to feeling depressed, sad or blue. Over the last 2 weeks, how often have you been bothered by any of the following problems? Please circle the answer that best represents how you have been.

	Not at all	Several days	More than half the days	Nearly every day
1. Little interest or pleasure in doing things	0	1	2	3
2. Feeling down, depressed or hopeless	0	1	2	3
3. Trouble falling asleep, staying asleep, or sleeping too much	0	1	2	3
4. Feeling tired or having little energy	0	1	2	3
5. Poor appetite or overeating	0	1	2	3
6. Feeling bad about yourself – or that you're a failure or have let yourself or your family down	0	1	2	3
7. Trouble concentrating on things, such as reading the newspaper or watching television	0	1	2	3
8. Moving or speaking so slowly that other people could have noticed. Or, the opposite – being so fidgety or restless that you have been moving around a lot more than usual	0	1	2	3
9. Thoughts that you would be better off dead or of hurting yourself in some way	0	1	2	3

Screening 3

	No	Yes
Have you used cannabis/marijuana-containing products (e.g., vapes, concentrates, edibles, joints, blunts, pills, oils, capsules, sprays, creams, K1, spice) or related products (synthetic cannabis) in the last 6 months?	0	1

Screening 4

	No	Yes
Have you used any tobacco (e.g., cigarettes, cigars, pipes, snuff, snus, chew, hooka) or nicotine-containing products (e.g., electronic-cigarettes, vapes, pouches, gum, lozenges, inhaler, nasal spray) in the last 6 months?	0	1

Screening 5

The following questions relate to your sleep habits. Please circle the best answer which you think represents your typical sleep habits over the recent past.

	5-6 hours	6-7 hours	7-8 hours	8-9 hours	more than 9 hours
1. During the recent past, how many hours of actual sleep did you get at night? (This may be different than the number of hours you spent in bed.)	4	3	2	1	0

	very satisfied	somewhat satisfied	neither satisfied nor dissatisfied	somewhat dissatisfied	very dissatisfied
2. How satisfied/dissatisfied are you with the quality of your sleep?	0	1	2	3	4

	15 minutes or less	16-30 minutes	31-60 minutes	longer than 60 minutes
3. During the recent past, how long has it usually taken you to fall asleep each night?	0	1	2	3

	never	once or twice per week	three or four times per week	five to seven times per week
4. How often do you have trouble staying asleep?	0	1	2	3
5. During the recent past, how often have you taken medicine to help you sleep (prescribed or over-the-counter)?	0	1	2	3

Screening 6

On how many of the past 7 days...

	0 days	1-2 days	3-5 days	6-7 days
1. Have you been deliberately trying to limit the amount of food you eat to influence your weight or shape (whether or not you have succeeded)?	0	1	2	3
2. Have you gone for long periods of time (e.g., 8 or more waking hours) without eating anything at all in order to influence your weight or shape?	0	1	2	3
3. Has thinking about food, eating or calories made it very difficult to concentrate on things you are interested in (such as working, following a conversation or reading)?	0	1	2	3
4. Has thinking about your weight or shape made it very difficult to concentrate on things you are interested in (such as working, following a conversation or reading)?	0	1	2	3
5. Have you had a definite fear that you might gain weight?	0	1	2	3
6. Have you had a strong desire to lose weight?	0	1	2	3
7. Have you tried to control your weight or shape by making yourself sick (vomit) or taking laxatives?	0	1	2	3
8. Have you exercised in a driven or compulsive way as a means of controlling your weight, shape or body fat, or to burn off calories?	0	1	2	3
9. Have you had a sense of having lost control over your eating (at the time that you were eating)?	0	1	2	3
10. On how many of these days (i.e. days on which you had a sense of having lost control over your eating) did you eat what other people would regard as an unusually large amount of food in one go?	0	1	2	3

Over the past 7 days...

	Not at all	Slightly	Moderately	Markedly
11. Has your weight or shape influenced how you think about (judge) yourself as a person?	0	1	2	3
12. How dissatisfied have you been with your weight or shape?	0	1	2	3

Screening 7

The following questions are about alcohol use. Please respond to each question by circling the number from '0' to '4' that represents your alcohol use.

	Never	Monthly or less	2-4 times a month	2-3 times a week	4 or more times a week
1. How often do you have a drink containing alcohol?	0 (skip to question 9)	1	2	3	4

	1-2	3-4	5-6	7-9	10 or more
2. How many standard drinks containing alcohol do you have on a typical day when you drink?	0	1	2	3	4

	Never	Less than monthly	Monthly	Weekly	Daily or almost daily
3. How often do you have six or more drinks on one occasion?	0	1	2	3	4
4. How often during the last year have you found that you were not able to stop drinking once you had started?	0	1	2	3	4
5. How often during the last year have you failed to do what was normally expected from you because of drinking?	0	1	2	3	4
6. How often during the last year have you needed a first drink in the morning to get yourself going after a heavy drinking session?	0	1	2	3	4
7. How often during the last year have you had a feeling of guilt or remorse after drinking?	0	1	2	3	4
8. How often during the last year have you been unable to remember what happened the night before because you had been drinking?	0	1	2	3	4

	No	Yes, but not in the last year	Yes, during the last year
9. Have you or someone else been injured as a result of your drinking?	0	2	4
10. Has a relative or friend or a doctor or another health worker been concerned about your drinking or suggested you cut down?	0	2	4

ATHLETE'S NAME: ....

ATHLETE'S ID NUMBER: ....

DATE: ....

ATHLETE FORM 2

Screening 8

Sometimes things happen to people that are unusually or especially frightening, horrible, or traumatic. For example, a serious accident or fire, a physical or sexual assault or abuse, an earthquake or flood, a war, seeing someone be killed or seriously injured, or having a loved one die through homicide or suicide. Please respond to the following question by circling 'yes' or 'no'.

Have you ever experienced this kind of an event?	No	Yes

If yes, did the event occur inside or outside of sport?	Inside	Outside	Both

If you have not experienced one or more of these events, then stop here with Screening 8 and please go to Screening 9.
If you have experienced an event or events like this, please circle your answer to the following 5 questions.

	No	Yes
1. In the past month, have you had nightmares about the event(s) or thought about the event(s) when you did not want to?	0	1
2. In the past month, have you tried hard not to think about the event(s) or went out of your way to avoid situations that reminded you of the event(s)?	0	1
3. In the past month, have you been constantly on guard, watchful, or easily startled?	0	1
4. In the past month, have you felt numb or detached from people, activities, or your surroundings?	0	1
5. In the past month, have you felt guilty or unable to stop blaming yourself or others for the event(s) or any problems the events may have caused?	0	1

Screening 9

The following questions are about drug(s) use in the last 3 months. Please respond to each question by circling 'yes' or 'no'. When thinking about drug use consider legal ones like caffeine or nicotine, illicit/illegal drugs (including cannabis even if legal in your state/country) and prescription medications used in ways other than prescribed (i.e., higher dosages; different ways of taking them, i.e., crushing/sniffing, injecting). Do NOT include alcohol in these responses.

	No	Yes
1. In the last three months, have you felt you should cut down or stop using drugs?	0	1
2. In the last three months, has anyone annoyed you or gotten on your nerves by telling you to cut down or stop using drugs?	0	1
3. In the last three months, have you felt guilty or bad about how much you use drugs?	0	1
4. In the last three months, have you been waking up wanting to use drugs?	0	1

In the last 3 months, which drug(s) or substance(s) listed below caused concerns or problems in your life? Concerns may include drug-related stress, depression, insomnia, financial strain, relationship conflict, heavy use/overdose, cravings, withdrawal, blackouts, flashbacks, fights, arrests, missed work, and/or medical problems like hepatitis, seizures or weight loss. Please circle all that apply.

None	Caffeine	Tobacco/Nicotine	Cannabis/Marijuana
Synthetic Cannabis (e.g., K2, Spice)	Prescription Stimulants (e.g., ADD, ADHD Medications)	Prescription Opioids-Pain (e.g., oxycodone, hydrocodone)	Other – Specify

ATHLETE'S NAME: ....

ATHLETE'S ID NUMBER: ....

DATE: ....

ATHLETE FORM 3

Screening 10

Please circle the answer that best describes how you have felt and conducted yourself over the past 6 months.

	Never	Rarely	Sometimes	Often	Very often
1. How often do you have trouble wrapping up the final details of a project, once the challenging parts have been done?	0	0	1	1	1
2. How often do you have difficulty getting things in order when you have to do a task that requires organisation?	0	0	1	1	1
3. How often do you have problems remembering appointments or obligations?	0	0	1	1	1
4. When you have a task that requires a lot of thought, how often do you avoid or delay getting started?	0	0	0	1	1
5. How often do you fidget or squirm with your hands or feet when you have to sit down for a long time?	0	0	0	1	1
6. How often do you feel overly active and compelled to do things, like you were driven by a motor?	0	0	0	1	1

Screening 11

Please respond to each question by circling 'yes' or 'no'.

	No	Yes
1. Has there ever been a period of time when you were not your usual self and... ...you felt so good or so hyper that other people thought you were not your normal self or you were so hyper that you got into trouble?	0	1
...you were so irritable that you shouted at people or started fights or arguments?	0	1
...you felt much more self-confident than usual?	0	1
...you got much less sleep than usual and found you didn't really miss it?	0	1
...you were much more talkative or spoke faster than usual?	0	1
...thoughts raced through your head or you couldn't slow your mind down?	0	1
...you were so easily distracted by things around you that you had trouble concentrating or staying on track?	0	1
...you had much more energy than usual?	0	1
...you were much more active or did many more things than usual?	0	1
...you were much more social or outgoing than usual, for example, you telephoned friends in the middle of the night?	0	1
...you were much more interested in sex than usual?	0	1
...you did things that were unusual for you or that other people might have thought were excessive, foolish, or risky?	0	1
...spending money got you or your family in trouble?	0	1
2. If you checked YES to more than one of the above, have several of these ever happened during the same period of time? Please check 1 response only.	0	1

	No problem	Minor problem	Moderate problem	Serious problem
3. How much of a problem did any of these cause you – like being able to work; having family, money, or legal troubles; getting into arguments or fights? Please check 1 response only.	0	0	1	1

Screening 11 (continued)

	No	Yes
4. Have any of your blood relatives (i.e., children, siblings, parents, grandparents, aunts, uncles) had manic-depressive illness or bipolar disorder?	0	1
5. Has a health professional ever told you that you have manic-depressive illness or bipolar disorder?	0	1

Screening 12

Please circle the answer that best represents how you have been feeling towards gambling in the last 12 months.

	Never	Sometimes	Most of the time	Almost always
1. Have you bet more than you could really afford to lose?	0	1	2	3
2. Have you needed to gamble with larger amounts of money to get the same feeling of excitement?	0	1	2	3
3. When you gambled, did you go back another day to try to win back the money you lost?	0	1	2	3
4. Have you borrowed money or sold anything to get money to gamble?	0	1	2	3
5. Have you felt that you might have a problem with gambling?	0	1	2	3
6. Has gambling caused you any health problems, including stress or anxiety?	0	1	2	3
7. Have people criticised your betting or told you that you had a gambling problem, regardless of whether or not you thought it was true?	0	1	2	3
8. Has gambling caused any financial problems for you or your household?	0	1	2	3
9. Have you felt guilty about the way you gamble or what happens when you gamble?	0	1	2	3

Screening 13

1. Have you lost someone significant to you?	No	Yes

2. How many months has it been since your significant other died?	Months

For each item below, please circle how you currently feel? Since the death, or as a result of the death...

	Not at all	Slightly	Somewhat	Quite a bit	Overwhelmingly
3. Do you feel yourself longing or yearning for the person who died?	1	2	3	4	5
4. Do you have trouble doing the things you normally do because you are thinking so much about the person who died?	1	2	3	4	5
5. Do you feel confused about your role in life or feel like you don't know who you are anymore (i.e., feeling like that a part of you has died)?	1	2	3	4	5
6. Do you have trouble believing that the person who died is really gone?	1	2	3	4	5
7. Do you avoid reminders that the person who died is really gone?	1	2	3	4	5
8. Do you feel emotional pain (e.g., anger, bitterness, sorrow) related to the death?	1	2	3	4	5
9. Do you feel that you have trouble re-engaging in life (e.g., problems engaging with friends, pursuing interests, planning for the future)?	1	2	3	4	5
10. Do you feel emotionally numb or detached from others?	1	2	3	4	5
11. Do you feel that life is meaningless without the person who died?	1	2	3	4	5
12. Do you feel alone or lonely without the deceased?	1	2	3	4	5

Screening 13 (continued)

13. Have the symptoms above caused significant impairment in social, occupational, or other important areas of functioning?	No	Yes

Screening 14

The following statements refer to experiences that many people have in their everyday lives.
Please circle the answer that best describes how much that experience has distressed or bothered you during the past month.

	Not at all	A little	Moderately	A lot	Extremely
1. I have saved up so many things that they get in the way.	0	1	2	3	4
2. I check things more often than necessary.	0	1	2	3	4
3. I get upset if objects are not arranged properly.	0	1	2	3	4
4. I feel compelled to count while I am doing things.	0	1	2	3	4
5. I find it difficult to touch an object when I know it has been touched by strangers or certain people.	0	1	2	3	4
6. I find it difficult to control my own thoughts.	0	1	2	3	4
7. I collect things I don't need.	0	1	2	3	4
8. I repeatedly check doors, windows, drawers, etc.	0	1	2	3	4
9. I get upset if others change the way I have arranged things.	0	1	2	3	4
10. I feel I have to repeat certain numbers.	0	1	2	3	4
11. I sometimes have to wash or clean myself simply because I feel contaminated.	0	1	2	3	4
12. I am upset by unpleasant thoughts that come into my mind against my will.	0	1	2	3	4
13. I avoid throwing things away because I am afraid I might need them later.	0	1	2	3	4
14. I repeatedly check gas and water taps and light switches after turning them off.	0	1	2	3	4
15. I need things to be arranged in a particular order.	0	1	2	3	4
16. I feel that there are good and bad numbers.	0	1	2	3	4
17. I wash my hands more often and longer than necessary.	0	1	2	3	4
18. I frequently get nasty thoughts and have difficulty in getting rid of them.	0	1	2	3	4

Screening 15

Please circle the answer that best represents how you are feeling.

			If TRUE: how much distress did you experience?			
	True	False	None	Mild	Moderate	Severe
1. I feel uninterested in the things I used to enjoy.	☐	☐	0	1	2	3
2. I often seem to live through events exactly as they happened before (déjà vu).	☐	☐	0	1	2	3
3. I sometimes smell or taste things that other people can't smell or taste.	☐	☐	0	1	2	3
4. I often hear unusual sounds like banging, clicking, hissing, clapping or ringing in my ears.	☐	☐	0	1	2	3
5. I have been confused at times whether something I experienced was real or imaginary.	☐	☐	0	1	2	3
6. When I look at a person, or look at myself in a mirror, I have seen the face change right before my eyes.	☐	☐	0	1	2	3
7. I get extremely anxious when meeting people for the first time.	☐	☐	0	1	2	3
8. I have seen things that other people apparently can't see.	☐	☐	0	1	2	3

Screening 15 (continued)

	True	False	If TRUE: how much distress did you experience? None	Mild	Moderate	Severe
9. My thoughts are sometimes so strong that I can almost hear them.	☐	☐	0	1	2	3
10. I sometimes see special meanings in advertisements, shop windows, or in the way things are arranged around me.	☐	☐	0	1	2	3
11. Sometimes I have felt that I'm not in control of my own ideas or thoughts.	☐	☐	0	1	2	3
12. Sometimes I feel suddenly distracted by distant sounds that I am not normally aware of.	☐	☐	0	1	2	3
13. I have heard things other people can't hear like voices of people whispering or talking.	☐	☐	0	1	2	3
14. I often feel that others have it in for me.	☐	☐	0	1	2	3
15. I have had the sense that some person or force is around me, even though I could not see anyone.	☐	☐	0	1	2	3
16. I feel that parts of my body have changed in some way, or that parts of my body are working differently than before.	☐	☐	0	1	2	3
