# Supplementary material for: International Olympic Committee Sport Mental Health Assessment Tool 2 (IOC SMHAT2): development, content validity and feasibility
Source: Br J Sports Med. 2026 Jul 16;60(15):e111408. doi: 10.1136/bjsports-2025-111408 (PMC13479680; doi:10.1136/bjsports-2025-111408)
Supplement: Supplementary file 3 [file bjsports-60-15-s005.pdf]

Online supplementary material 5

Measurement properties of the questionnaires embedded in Step 1 and Step 2 of the International Olympic Committee Sport Mental Health Assessment Tool 2 (IOC SMHAT2)

Mental health symptom cluster	Instrument	Number of items	Rating scale and recall period	Scoring	Reliability	Validity
Anxiety[21]	General Anxiety Disorder-7 (GAD-7)	7	4-points scale: from "not at all" (0) to "nearly every day" (3)<br><br>Recall period: last 2 weeks	Sum: from 0 to 21<br>Cut-off: >9<br><br>Severity:<br>Mild = 5-9<br>Moderate = 10-14<br>Severe: >14	Internal consistency: 0.92<br>Test-retest reliability: 0.83	Area under ROC curve >0.9<br>Sensitivity: 89<br>Specificity: 82
Depression[22,23]	Patient Health Questionnaire-9 (PHQ-9)	9	4-points scale: from "not at all" (0) to "nearly every day" (3)<br><br>Recall period: last 2 weeks	Sum: from 0 to 27<br>Cut-off: >9<br><br>Severity:<br>Mild = 5-9<br>Moderate = 10-14<br>Moderate severe = 15-19<br>Severe: >19	Internal consistency: 0.83-0.89	Area under ROC curve: 0.95<br>Sensitivity: 88<br>Specificity: 88

				**Need score 2 or 3 on one of first 2 questions to warrant treatment or treatment change*		
Sleep disturbance[24-26]	Athlete Sleep Screening Questionnaire (ASSQ)	16	Various scales dichotomous, 4-points scales or 5-points scales<br><br>Recall period: recent past	Sum: from 0-17<br>Cut-off: >7<br><br>Severity:<br>Mild: 5-7;<br>Moderate: 8-10<br>Severe: 11-17	Internal consistency: 0.74<br>Test-retest reliability: 0.86	Sensitivity: 81%<br>Specificity:93%<br>Positive predictive value: 87%<br>Negative predictive value: 90%
Disordered eating[27,28]	Eating Disorder Examination Questionnaire Short (EDE-QS)	12	4-points scale: from (0) to (3)<br><br>Recall period: past 7 days	Sum: from 0 to 36<br>Cut-off: >14	Internal consistency: 0.86-0.93<br>Test-retest reliability: 0.85-0.90	Area Under ROC Curve: 0.85-0.95
Alcohol misuse[29,30]	Alcohol Use Disorders Identification Test (AUDIT)	10	5-points and 3-points scales: from (0) to (4)<br><br>Recall period: past year	Sum: from 0 to 40<br>Cut-off: >7<br><br>Severity:<br>Hazardous or harmful consumption: 8-14	Internal consistency: 0.75-0.92<br>Test-retest reliability: 0.80-0.90	Area Under ROC Curve: 0.80-0.98<br>Sensitivity: 80-95%<br>Specificity 70-90%

				Moderate-severe alcohol use disorder: >14		
Post traumatic stress[31,32]	Primary Care Post Traumatic Stress Disorder Screen for DSM-5 (PC-PTSD-5)	5	Yes (1) or No (0)<br><br>Recall period: past month	Sum: from 0 to 5<br>Cut-off: >2	Internal consistency: 0.70-0.75<br>Test-retest reliability: 0.80	Area Under ROC Curve: 0.85-0.90<br>Sensitivity: 85-95%<br>Specificity 70-85%
Substance misuse[33-38]	Cutting down, Annoyance by criticism, Guilty feeling, and Eye-openers Adapted to Include Drugs (CAGE-AID)	4	Yes (1) or No (0)<br><br>Recall period: last 3 months	Sum: from 0 to 4<br>Cut-off: >1	Reliability: >0.9	Area Under ROC Curve: 0.80 for cannabis use; 0.99 for amphetamine use<br>Sensitivity: >79%<br>Specificity: >97%
