# Supplementary material for: International Olympic Committee Sport Mental Health Assessment Tool 2 (IOC SMHAT2): development, content validity and feasibility
Source: Br J Sports Med. 2026 Jul 16;60(15):e111408. doi: 10.1136/bjsports-2025-111408 (PMC13479680; doi:10.1136/bjsports-2025-111408)
Supplement: Supplementary file 4 [file bjsports-60-15-s006.pdf]

IOC SMHRT2

The International Olympic Committee Sport Mental Health Recognition Tool 2
Developed by the IOC Mental Health Working Group

International Olympic Committee

What is the IOC SMHRT2?

Sadness, anger, stress, irritability and anxiety are all normal parts of the human experience; however, if these problems persist for long periods of time or have a big impact on someone's sport career or daily life, it may indicate that the athlete is experiencing a mental health problem. As mental health problems are common in elite athletes, it remains essential to identify them as early as possible in order to refer the athlete for management and/or treatment for potential mental health problems in a timely manner.

Who should use the IOC SMHRT2?

The International Olympic Committee (IOC) Sport Mental Health Recognition Tool 2 (SMHRT2) can be used by athletes, coaches, family members and all other members of the athlete's entourage to recognise mental health problems but not to diagnose them. The IOC SMHRT2 presents a list of athlete experiences (thoughts, feelings, behaviours, physical changes) that could be indicative of mental health problems. If an athlete reports and/or displays these experiences and they are significant and/or persistent, you have an important role in encouraging the athlete to get the support needed as early as possible.

Why use the IOC SMHRT2?

The IOC SMHRT2 in its current form can be freely copied for distribution to individuals, teams, groups and organizations. Any revision requires the specific approval by the IOC MHWG while any translation should be reported to the IOC MHWG. The IOC SMHRT2 should not be re-branded or sold for commercial gain.

Further information and material on mental health in elite athletes has been developed by the IOC and can be found here.

COMMON EXPERIENCES OF MENTAL HEALTH PROBLEMS

1

Thoughts

Excessive self-criticism, low self-esteem, pessimism, hopelessness, problems with focus, concentration and memory.

Feelings

Irritability, anger, mood swings, sadness, extreme disappointment that you just cannot shake, depression, loneliness, emptiness, lack of passion and sense of purpose, lack of motivation.

Actions

Aggression, withdrawal from others / not going outside as much, being much more quiet than usual, unexpected drop in performance (e.g., in sport, school, work).

Physical changes

Low energy, poor sleep, changes in appetite, changes in weight and appearance, physical signs of harm by self or others including cuts and bruises, evidence of alcohol or other substance misuse (e.g., tremors, blood-shot eyes, change in pupil size, characteristic smell of marijuana or alcohol, slowed or poor coordination, injuries or arrests after using).

RED FLAGS

If an athlete (or you) experience or observe any of the following, seek immediate help.

- Comments related to harming self or others.
- Talking about feeling hopeless or so overwhelmed that you cannot function.
- Dramatic weight changes.
- Other highly uncharacteristic behaviours, emotions and appearances.
- An episode of overwhelming sudden onset of fear with marked physical symptoms such as sweating or shortness of breath that has never before been experienced or is different from prior episodes (could be a panic attack or another medical problem).

WHAT TO DO WHEN MENTAL HEALTH PROBLEMS OCCUR?

If you are observing mental health problems in an athlete, consider the following (depending on your role):

- Allow the athlete to tell their story.
- Be non-judgemental and understanding (e.g., with simple statements such as "I'm so sorry you are feeling this way; I'm glad you told me about it.").
- Know which resources are available and encourage help-seeking.
- Remember to look after yourself when assisting an athlete with mental health challenges.
- Consider what adjustments in your coaching approach might be needed to help an athlete's mental health while maintaining or creating a positive environment (e.g., recognising the need for positive feedback, encouragement and the right balance of challenge versus support).
- Consult with the medical team and discuss the possible adjustment of an athlete's training, in consultation with your technical staff.

If you personally are experiencing mental health problems:

Remember that seeking help is a sign of strength. Pay attention to what you are experiencing. Prioritise your mental health and life balance. Talk to someone you trust such as your coach, your parents, a friend and/or a teammate. Be open to advice and support. Consider seeking professional help.
