# Supplementary material for: International Olympic Committee Sport Mental Health Assessment Tool 2 (IOC SMHAT2): development, content validity and feasibility
Source: Br J Sports Med. 2026 Jul 16;60(15):e111408. doi: 10.1136/bjsports-2025-111408 (PMC13479680; doi:10.1136/bjsports-2025-111408)
Supplement: Supplementary file 5 [file bjsports-60-15-s001.docx]

**Online supplementary material 1**

**Questions for the interview with experienced users of the Sport Mental Health Assessment Tool 1 (SMHAT-1)**

1. Can you elaborate on the context in which you used or are using the IOC SMHAT-1?

[*e.g.*, country, athletes, period of use]

2. What are the facilitators you have experienced in your use of the IOC SMHAT-1?

3. What are the barriers you have experienced to using the IOC SMHAT-1?

4. The Sport Mental Health Assessment Tool 1 is based on a 3-step approach, namely step 1 as triage step using the APSQ, step 2 as extensive screening step using 6 validated questionnaires, and step 3 as interventional step. What would be your advice regarding these steps for the forthcoming IOC SMHAT-2?

5. The 3 Athletes’ forms in the SMHAT-1 include various validated questionnaires such as the GAD-7 and PHQ-9 for measuring various types of mental health symptoms or disorders. What would be your advice for these questionnaires or for consideration of other questionnaires for the IOC SMHAT2? Please be as specific as possible.

6. An important component of the IOC SMHAT-1 is the clinical assessment. That is, the questionnaires themselves do not provide a definite diagnosis. They must be followed up, if the questionnaire results call for it, with a clinical assessment with a medical professional. What would be your advice with regard to this clinical assessment for the forthcoming IOC SMHAT2?

7. Any final suggestions to add?
