# Supplementary material for: International Olympic Committee Sport Mental Health Assessment Tool 2 (IOC SMHAT2): development, content validity and feasibility
Source: Br J Sports Med. 2026 Jul 16;60(15):e111408. doi: 10.1136/bjsports-2025-111408 (PMC13479680; doi:10.1136/bjsports-2025-111408)
Supplement: Supplementary file 6 [file bjsports-60-15-s003.docx]

**Online supplementary material 3**

**Qualitative feedback related to the validity and feasibility of the International Olympic Committee Sport Mental Health Assessment Tool 2 (IOC SMHAT2)**

- Step 1 and 2 seems to be much easier than SMHAT-1. Step 3 requires a clinical or trained sports psychiatrist within the network, which is a different issue from the screening tool but needs to be considered in a variety of contexts.
- To streamline the administration, scoring, and interpretation of the test, we can incorporate an online or technology-based questionnaire. This approach allows for rapid result generation, eliminating the need for manual analysis. Given that we are working with elite athletes‚ whose time is highly valuable, and delivering immediate feedback will enhance the test's usability and make it more athlete friendly.
- Why not recommend M.I.N.I instead of the questionaries in step three? Much better than using the screening tools suggested.
- It is implementable, possible and doable because I am in a well-resourced setting. However, in a lower resourced setting and/or with athletes who have a limited amount of time, I believe this will be overly burdensome and difficult to implement. I would highly recommend developing a new sensitive screening test to make the SMHAT2 more feasible for broad use.
- It depends how easy to use the form is - if it is pen and paper, then it will be a problem. If it is an online form (not an online PDF), then that will be more straightforward to use.
- Using the IOC SMHAT2 with elite athletes in East Asia may require particular attention to cultural factors. Psychological distress is often expressed through somatic complaints, and stigma can reduce athlete willingness to disclose mental health symptoms. What may appear as diagnostic uncertainty or treatment non-response could, in fact, reflect cultural tendencies to minimize or normalize distress. Ensuring psychological safety during assessment, and when appropriate involving team/organization contexts, can enhance both feasibility and effectiveness of SMHAT2 implementation in this setting.
- Important note: Forms 2 and 3 are screening tools only and do not replace a comprehensive diagnostic evaluation, which must include a structured or semi-structured clinical interview such as the ADIS (Anxiety Disorders Interview Schedule) or the SCID (Structured Clinical Interview for DSM disorders), conducted by a qualified mental health professional.
- It is a long measure all together and I think it could be more targeted to athletes in some ways and more general in others (see my previous comments).
- Perhaps, with time there will be athletes customized short version questionnaire that will capture the core symptoms of mental disorders, that will be administered over a short time.
- I think the athlete should have a right to agree or not to complete SMHAT2 form 1 which even in its basic form is intrusive. Participation should be encouraged and promoted among elite athletes. For those who progress to form 2 and 3 referral pathways will have to be in place and will be a nightmare to organize and involve strong cooperation of NFs and the medical professionals
- I think this tool will be a valuable asset to physicians, coaches, and athletes
- I absolutely this this is feasible. Its comprehensive and lengthy which is great because its thorough and provides clinicians with clear direction around how to navigate potentially complex situations. Its comprehensiveness though could pose barriers to some in terms of time and resource constraints, but I don't think that's a reason to change anything about it.
- This tool may well form part of a more comprehensive approach to understanding mental health needs of individual athletes, but this tool inappropriately simplifies the complexity and need for comprehensive investigation by inappropriately medicalizing experiences that may well be a normal part of athlete and human for that matter life. While I do not disagree that individuals scoring certain scores may require intervention. I don’t believe that this is the primary way we should be promoting such enquiries
- I think there remains some gray area in when the screening tools for Athlete Form 3 would be used. The document indicates it is in cases of diagnostic uncertainty or further information is needed, but it still seems to me there may be areas where something, particularly problem gambling, may be missed.
- Our medical doctors don't do clinical interviews but screenings. They are not allowed or educated to set diagnoses. For that, the third step should be done by a clinical or mental health psychologist. I don't think that's clear in the interview now.
- Surprised at what is in phase 2.
- Very feasible to implement with the appropriate personnel, protocols, and resources for meaningful follow up.
- Nice to give approximate insights to users about time required in different scenarios
- I am answering this as a professional in a westernized country and wonder whether this is available in other languages and validated in other populations?
- I think some kind of training and standardization or even cultural relevance will be great .. it will be easiest to implement it across the globe
- "If there is automated scoring and can be distributed easily, then this could be feasible.
- Or, if we could re-arrange the form for our own use (e.g., adding screeners from form 3 to form 1)"
- The implementation is certainly feasible.
- Regarding step 3, I am curious. The language of the protocol suggests involving a mental health professional only when cases are severe, complex, diagnostically uncertain and/or unresponsive. I wonder if we shouldn't advocate for earlier involvement when possible.
- I think the SMATH-2 is great. There are a few points to discuss. At this point in time, I consider the SMATH-2 to be extremely important and very good.
- For the future, the question arises as to whether a medical history should also be included: Have you ever been in psychiatric or psychotherapeutic treatment in the past? Do you currently have mental health issues? Are you currently undergoing psychiatric or psychotherapeutic treatment?
- Consideration should be given to integrate more psychiatric staff into the diagnostic process in the future, thereby identifying complex cases and proposing a treatment option, particularly with regard to dimensional diagnosis and underlying personality structures.
- In order to use the paper version of the SMHAT2, it would take so much time to administer and review with athletes, as well as provide proper clinical assessment for those that may be directed to meet with a clinician. Many collegiate institutions do not have large staffs, or even in-house staff at all. Digitizing the tool is also large financial investment for many schools.
- The SMHAT2 is a helpful tool. But needs to be updated as indicated
- In my current setting, there are abundant resources/time/technology to feasibly implement this. In other settings, community based or private practices, there could be limitations to material or technology to assist, yet still feasible.
- To say a tool is easy to use will depend on the responses and feedback gained over time and the comprehension of the athletes, in my certain l would have to interpret the various sections which is not an easy task
- Sometimes hard to understand how to use it - for example the part of ticking boxes about the severity and complexity - are the boxes ticked for HIGH severity, what is the limit for ticking the box, one would merely think of “severity" as something scalable like 1-5 etc.
- Also very time-consuming, and at least in my job as a physician for the nation’s best athletes there is not enough time for screenings, which is very sad.
- The only part that is confusing is the grid with "severity, complexity, etc." After reading the directions, I am still not certain as to what needs to be ticked. "Severity" for example would indicate a need for a level (i.e. 1-10). Unless the intent is "severe," which would be a binary answer of yes/no.
